# The β3‐integrin endothelial adhesome regulates microtubule‐dependent cell migration

**DOI:** 10.15252/embr.201744578

**Published:** 2018-05-24

**Authors:** Samuel J Atkinson, Aleksander M Gontarczyk, Abdullah AA Alghamdi, Tim S Ellison, Robert T Johnson, Wesley J Fowler, Benjamin M Kirkup, Bernardo C Silva, Bronwen E Harry, Jochen G Schneider, Katherine N Weilbaecher, Mette M Mogensen, Mark D Bass, Maddy Parsons, Dylan R Edwards, Stephen D Robinson

**Affiliations:** ^1^ School of Biological Sciences Norwich Research Park University of East Anglia Norwich UK; ^2^ Luxembourg Center for Systems Biomedicine (LCSB) Luxembourg & Saarland University Medical Center Internal Medicine II University of Luxembourg Homburg Germany; ^3^ Centre Hospitalier Emily Mayrisch Esch Luxembourg; ^4^ Division of Molecular Oncology Department of Internal Medicine Washington University in St Louis St. Louis MO USA; ^5^ Department of Biomedical Science Centre for Membrane Interactions and Dynamics University of Sheffield Sheffield UK; ^6^ Randall Division of Cell and Molecular Biophysics King's College London New Hunt's House, Guys Campus London UK; ^7^ Faculty of Medicine and Health Sciences Norwich Research Park University of East Anglia Norwich UK

**Keywords:** adhesome, endothelial, integrins, microtubules, Cell Adhesion, Polarity & Cytoskeleton, Methods & Resources, Vascular Biology & Angiogenesis

## Abstract

Integrin β3 is seen as a key anti‐angiogenic target for cancer treatment due to its expression on neovasculature, but the role it plays in the process is complex; whether it is pro‐ or anti‐angiogenic depends on the context in which it is expressed. To understand precisely β3's role in regulating integrin adhesion complexes in endothelial cells, we characterised, by mass spectrometry, the β3‐dependent adhesome. We show that depletion of β3‐integrin in this cell type leads to changes in microtubule behaviour that control cell migration. β3‐integrin regulates microtubule stability in endothelial cells through Rcc2/Anxa2‐driven control of active Rac1 localisation. Our findings reveal that angiogenic processes, both *in vitro* and *in vivo*, are more sensitive to microtubule targeting agents when β3‐integrin levels are reduced.

## Introduction

Angiogenesis, the formation of new blood vessels from those that already exist, plays an essential role in tumour growth 1. As such, targeting angiogenesis is seen as crucial in many anti‐cancer strategies 2. Therapies directed against vascular endothelial growth factor (VEGF) and its major receptor, VEGF‐receptor‐2 (VEGFR2), whilst effective in a number of cancers, are not without side‐effects due to the role this signalling pathway plays in vascular homeostasis 3. Fibronectin (FN)‐binding endothelial integrins, especially αvβ3‐ and α5β1‐integrins, have emerged as alternative anti‐angiogenic targets because of their expression in neovasculature 4, 5. However, neither global nor conditional knockouts of these integrins block tumour angiogenesis in the long term 6, 7, 8, and clinical trials of blocking antibodies and peptides directed against these extracellular matrix (ECM) receptors have been disappointing 9, 10. To gain novel insight into how αvβ3‐integrin regulates outside‐in signal transmission 11, we have undertaken an unbiased analysis of the molecular composition of the mature endothelial adhesome and profiled changes that occur when β3‐integrin expression is manipulated. In so doing, we have uncovered β3‐integrin‐dependent changes in microtubule behaviour that regulate cell migration.

## Results and Discussion

**Figure 1:**
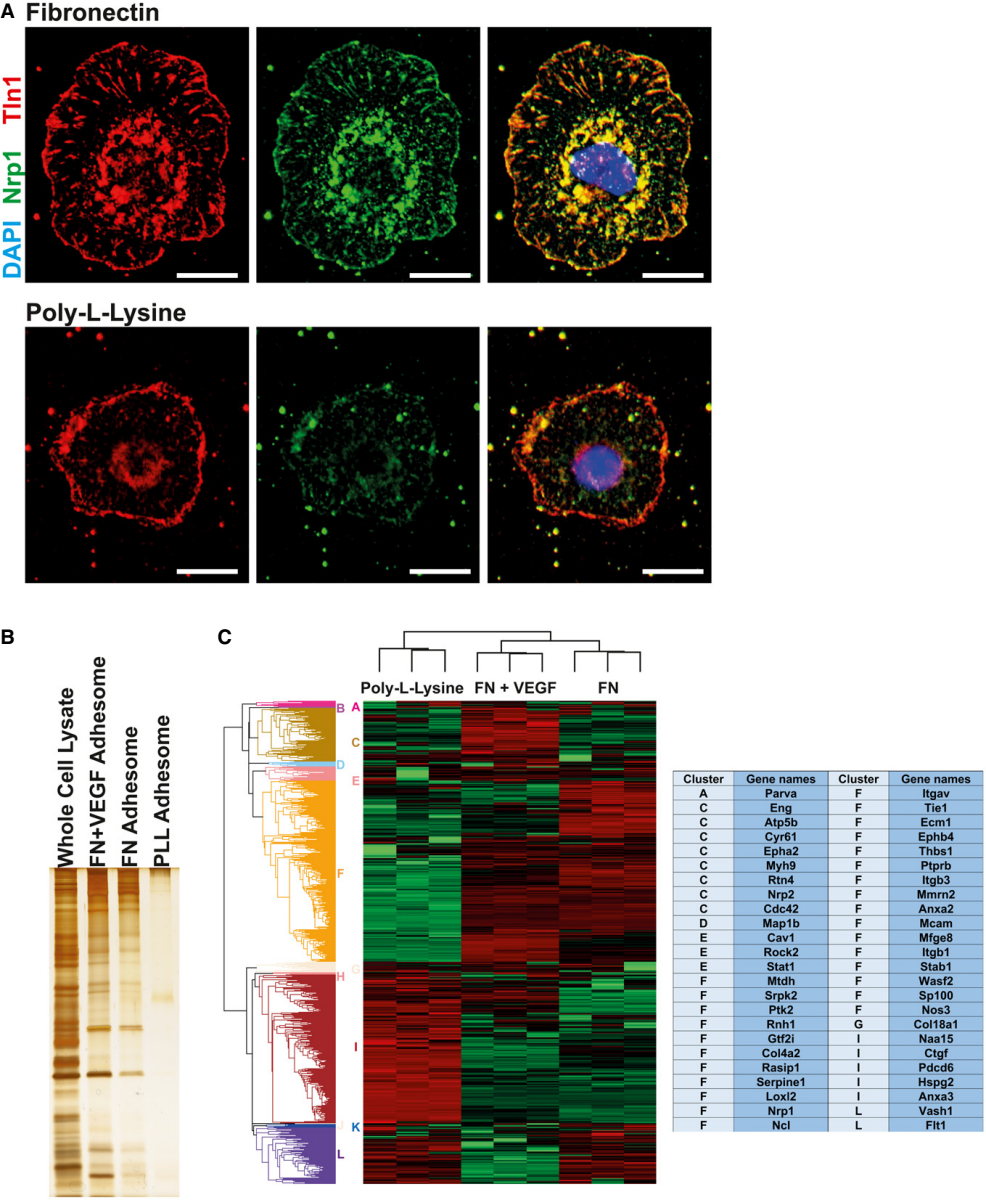
Defining the FN endothelial adhesome WT ECs were adhered to fibronectin‐ (top row) or poly‐l‐lysine (bottom row)‐coated coverslips for 90 min before fixing and immunostaining for neuropilin‐1 (Nrp1, green) and talin‐1 (Tln1, red) along with a nuclear stain (DAPI, blue). Scale bar = 10 μm.An example silver stain used in the quality control of adhesome samples. Adhesome enrichment was carried out on 6 × 10^6^ WT ECs on fibronectin (FN), fibronectin with VEGF (FN + VEGF) or poly‐l‐lysine (PLL) before acetone precipitation. After resuspension, samples were run on a SDS–PAGE gel along with a whole cell lysate control and silver‐stained.Triplicate adhesome samples from WT ECs adhered on fibronectin (FN), fibronectin with VEGF (FN + VEGF) or poly‐l‐lysine were sent for quantitative mass spec analysis. Label‐free quantification was carried out using MaxQuant followed by analysis in Perseus. Unsupervised hierarchical clustering (Euclidian distance calculation) was carried out with red showing highly abundant proteins and green showing low abundance proteins. Twelve significant clusters were automatically identified using a distance threshold of 3.34 and labelled as A–L. Angiogenesis‐associated proteins were identified using GOBP annotations (GO:0001525, GO:0002040, GO:0002042, GO:0016525, GO:0045765, GO:0045766) and are displayed in the table along with their associated cluster.

The isolation and analysis of integrin adhesion complexes (IACs) by mass spectrometry (MS) is difficult because of the low affinity and transient nature of the molecular interactions occurring at these sites. However, using cell‐permeant chemical cross‐linkers improves recovery of IAC proteins bound to either FN‐coated microbeads 12 or plastic dishes 13. These advances have led to the characterisation of IACs from a number of cell types. Whilst a core consensus adhesome (the network of structural and signalling proteins involved in regulating cell‐matrix adhesion 14) can be defined 12, the composition and stoichiometry of the meta‐adhesome depend on the cell type being analysed, the integrin‐receptor repertoire expressed by that cell type, and any imposed experimental conditions. To examine the composition of the endothelial adhesome, we isolated lung microvascular endothelial cells (ECs) from C57BL6/129Sv mixed background mice and immortalised them with polyoma‐middle‐T‐antigen by retroviral transduction 15. As our main interest was in establishing how β3‐integrin influences the endothelial adhesome, we adhered cells to FN for 90 min, which allows β3‐rich (mature) focal adhesions (FAs) to form 16. To distinguish integrin‐mediated recruitment of proteins from non‐specific background, we also plated cells on poly‐l‐lysine (PLL) as a negative control (adhesion to PLL does not depend on integrins). Visualisation of neuropilin‐1 staining in whole cells showed that this protein, which we previously demonstrated is present in the mature EC adhesome 17, co‐localises with talin‐1 in FAs when cells are plated on FN, but not PLL (Fig 1A). For all proteomics experiments, we cross‐linked FAs using the cell‐permeant and reversible cross‐linkers DPDPB and DSP (see Materials and Methods) for 5 min. Cells were lysed and subjected to a high shear flow water wash to remove non‐cross‐linked material. Crosslinking was reversed, and samples were precipitated and concentrated for analyses. Prior to MS, samples were quality‐controlled by SDS–PAGE and silver staining to ensure efficient removal of non‐cross‐linked material had occurred (Fig 1B).

Label‐free proteomic analyses of the FN + VEGF, FN, and PLL adhesomes (Fig 1C; Table EV1) initially detected and quantified 1,468 proteins. Stringent filtering, requiring proteins to be detected in all three repeats of at least one condition, left 1,064 proteins—a high confidence dataset that was used to define the endothelial adhesome. Hierarchical clustering based on average Euclidian distance identified 12 clusters (A–L), which could be considered VEGF‐enriched (A–C), FN‐enriched (D–F) and PLL‐enriched (G–L) proteins. Fisher's exact test enrichment analysis was carried out to identify which pathway, process or component proteins within these clusters belong to using Gene Ontology annotations. Cell projection (GOCC, *P* = 8.62 × 10^−5^) and microtubule (GOCC, *P* = 1.6 × 10^−4^) categories were significantly enriched when cells were treated with growth factor, suggesting they are important in VEGF‐mediated processes. Leucocyte trans‐endothelial migration (KEGG, *P* = 9.71 × 10^−5^) proteins were enriched in the FN adhesome, but not in the VEGF‐stimulated adhesome, suggesting our cells represent quiescent vasculature without VEGF stimulation. This same category contains many endothelial‐specific proteins (e.g. VE‐cadherin, Cdh5), further confirming that the cells have an endothelial identity. Focal adhesion (KEGG, *P* = 9.31 × 10^−7^) proteins were enriched in the FN adhesome but depleted in the PLL adhesome, confirming the success of the adhesome enrichment process, MS and downstream analysis. Other adhesion/migration‐associated categories, focal adhesion (GOCC, *P* = 5.99 × 10^−5^), cell projection (GOCC, *P* = 3.03 × 10^−5^), cell adhesion (GOBP, *P* = 1.61 × 10^−6^) and lamellipodium (GOCC, *P* = 1.38 × 10^−4^), were depleted in the PLL adhesome.

**Figure 2:**
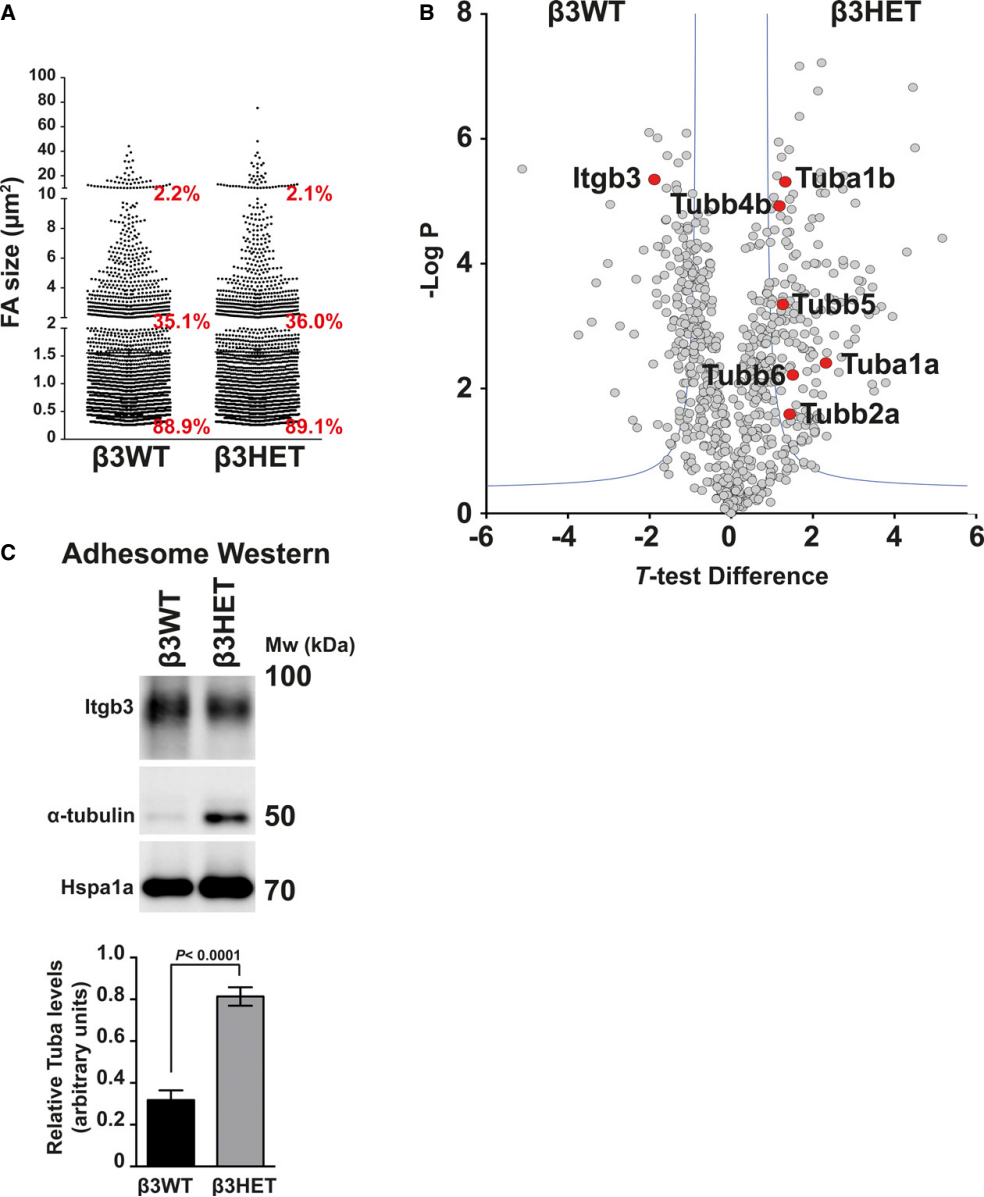
Analysis of the β3‐integrin‐dependent adhesome Distribution of adhesion size classes (0–2 μm; 2–10 μm; > 10 μm) in β3WT versus β3HET endothelial cells (*n* = 1,400 FAs per genotype, from two independent experiments).Visual representation of the significance analysis of microarrays (SAM) method as a volcano plot for β3WT and β3HET samples (*n* = 3). *t*‐Test difference is plotted against −log of the *P*‐value. The blue lines show the cut‐off for significance as defined by the SAM. Integrin‐β3 (Itgb3) as well as all detected tubulins (Tub) have been highlighted as red points.Adhesome samples from β3WT and β3HET endothelial cells adhered to fibronectin. Samples were Western‐blotted for integrin‐β3 (Itgb3), α‐tubulin and heat‐shock protein 70 (Hspa1a). Blot shown is representative of the five individual experiments that are quantified in the bar graph below. Bars = mean (±SEM) relative α‐tubulin levels normalised to Hspa1a levels. Significant differences between means were evaluated by unpaired two‐tailed Student's *t‐*test.

To test the consequences of excluding β3‐integrin from the EC adhesome, we decided to profile changes in β3‐heterozygous (β3HET) ECs, which carry one wild‐type allele of β3‐integrin and one knockout allele. These cells express 50% wild‐type levels of β3‐integrin. As in previous studies, we decided to use β3HET cells for these initial analyses, rather than β3‐integrin knockout (β3NULL) cells, hypothesising this would circumvent potential developmental changes arising from the complete loss of the protein, which we felt might confound quantitative interpretations of the EC adhesome; we have shown these cells are a good model for studying the role of αvβ3‐integrin in cell migration, whilst evading changes arising from the complete loss of the integrin on both alleles (e.g. up‐regulated total VEGFR2 expression) 17. Both wild‐type (β3WT) and β3HET ECs adhere equally to saturating concentrations (10 μg ml^−1^) of FN (see Ellison *et al* 17). To compare the size distribution of FAs between β3WT and β3HET ECs (which might affect the stoichiometry of components in the adhesome), we seeded cells for 90 min on FN, immunostained for paxillin, and measured FA area; we noted no differences in the percentage of FA size distributions between the two genotypes (Fig 2A). Therefore, MS analyses comparing the adhesome between β3WT and β3HET ECs were performed (Fig 2B; Table EV2). Enrichment analysis showed a depletion of cytoskeletal components (GOCC, *P* = 4.73 × 10^−5^) in the β3WT adhesome when compared with the β3HET adhesome, despite the enrichment of adhesion/migration‐associated categories previously noted in the FN adhesome of β3WT ECs (Fig 1C). Whilst a majority of individual FA components in the mature adhesome do not change upon β3‐integrin depletion, downstream connections to cytoskeletal components do. We took a particular interest in microtubules (MTs) because by SAM analysis all detected tubulins were significantly up‐regulated in the β3HET adhesome. To confirm this finding by other means, we probed Western blots for α‐tubulin and showed a significant increase in FA‐enriched samples from β3HET cells compared with β3WT cells (Fig 2C).

**Figure 3:**
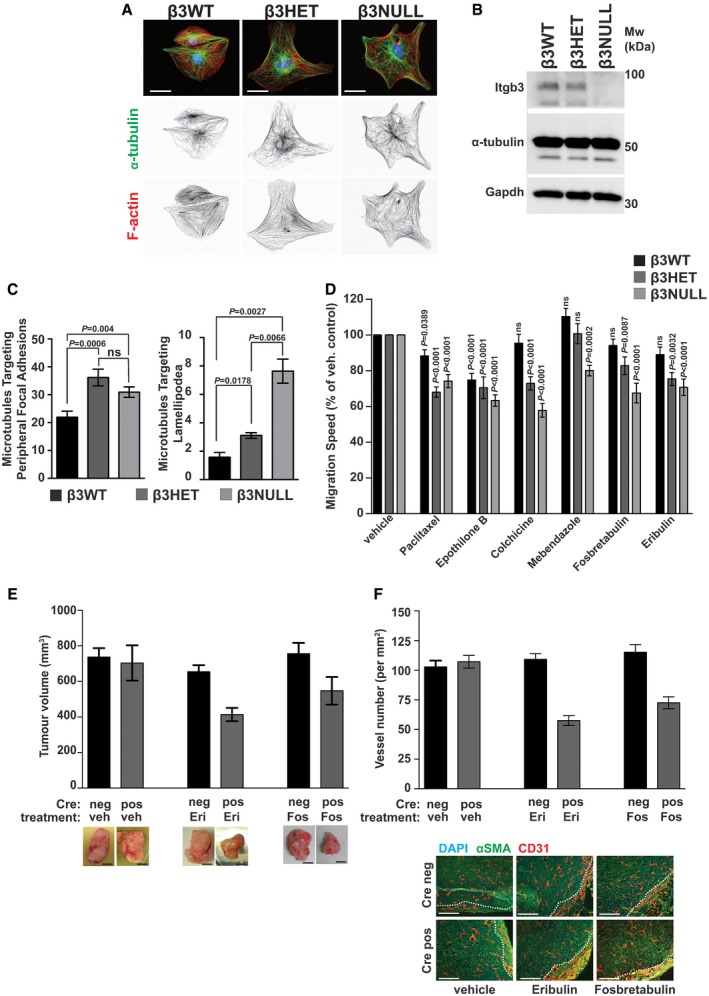
Analysis of microtubules in β3WT, β3HET and β3NULL endothelial cells β3WT, β3HET and β3NULL endothelial cells were adhered to fibronectin‐coated coverslips for 90 min before being PHEMO fixed and immunostained for α‐tubulin (green). Nuclear (DAPI, blue) and phalloidin (F‐actin, red) stains were also used. Inverted black and white images of α‐tubulin and F‐actin are shown below the three‐colour overlays. Scale bar = 10 μm.β3WT, β3HET and β3NULL endothelial cells were adhered to fibronectin for 90 min before being lysed and Western‐blotted for integrin‐β3 (Itgb3), α‐tubulin and Gapdh (as a loading control).
*Left:* β3WT, β3HET and β3NULL endothelial cells were adhered to fibronectin‐coated coverslips for 90 min before being methanol (−20°C) fixed and immunostained for α‐tubulin and talin‐1. The number of microtubules that terminated (overlapping staining) at a talin‐1 containing focal adhesion was counted for each genotype (*n* = 15 cells per genotype, from three independent experiments). *Right:* β3 WT, HET and NULL ECS were transfected with paxillin‐GFP and left to recover overnight. The cells were then adhered to fibronectin‐coated coverslips and allowed to recover for 3 h before being treated with 100 nM SiRTubulin and 1 μM verapamil overnight. The next day, fresh media containing SiRTubulin and verapamil (same dose) were added and cells were imaged every minute for 30 min (*n* = 3 cells per genotype, from three independent experiments). Areas of adhesive fronts were assessed by measuring the growth of paxillin–GFP‐positive areas between the 1^st^ and 30^th^ image. The number of microtubules that entered the adhesive front was quantified to give the number of microtubules entering lamellipodia relative to the area of adhesive fronts for each cell. Significant differences between means were evaluated by unpaired two‐tailed Student's *t‐*test. Error bars are ±SEM.β3WT, β3HET and β3NULL endothelial cells were adhered to fibronectin overnight. Migration speed of individual cells was measured over 15 h using the MTrackJ plugin for ImageJ under the influence of the indicated MTA. Migration speeds are shown as a percentage of the speed of the corresponding genotype under DMSO (vehicle) treatment (*n* ≥ 46 cells per genotype, from four independent experiments). Significant differences between means were evaluated by unpaired two‐tailed Student's *t‐*test. Error bars are ±SEM.β3flox/flox Tie1Cre‐positive (pos) and Cre‐negative (neg) animals were injected subcutaneously with 1 × 10^6^ CMT19T lung carcinoma cells and then treated with vehicle (veh), or Eribulin (Eri). Bar graph shows mean (±SEM) tumour volumes (*n* ≥ 6; from two to three independent experiments for each treatment condition) at the end of the experiment. Micrographs (below) show representative tumours. Scale bars = 5 mm.After excision, tumours from β3flox/flox Tie1Cre‐positive (pos) and Cre‐negative (neg) animals were processed and CD31 staining was assessed in vessel hot spots (see Materials and Methods) to measure vascular density. Bars = mean (±SEM) vessel number per mm^2^ (*n* = 5 sections from each genotype, taken over two to three independent experiments for each treatment condition). Micrographs (below) show representative images of sections stained for alpha‐smooth muscle actin (αSMA, green), CD31 (red), DAPI (blue). Dotted white line indicates border of tumour and surrounding connective tissue. Scale bars = 100 μm.

**Figure EV1:**
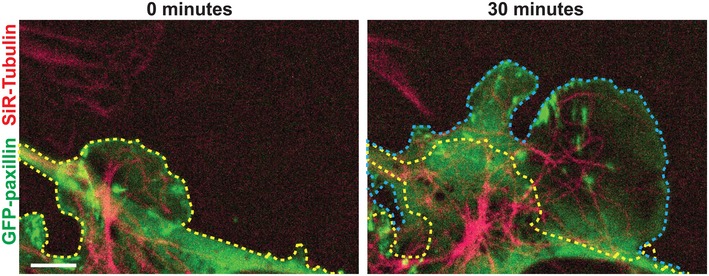
Measuring microtubule extension into lamellipodium Schematic demonstrating how Fig 3C (right) was calculated. The yellow line indicates the edge of Pxn‐GFP (green)‐positive areas at 0 min, and the blue line indicates the edge at the end of 30 min. Microtubules were labelled red with SiRTubulin. Scale bar = 5 μm.

Our findings intimated that αvβ3‐integrin drives MT localisation away from FAs. To increase the power of our downstream mechanistic analyses, we felt it was appropriate to now also include β3NULL ECs in our studies. We examined MT organisation in β3WT, β3HET and β3NULL ECs by immunolabelling for α‐tubulin in whole cells (Fig 3A). No gross changes in cell microtubule arrays were observed. Furthermore, total cellular levels of α‐tubulin were similar across all three genotypes (Fig 3B). However, co‐localisation of MTs at peripheral FAs was greater in β3HET and β3NULL ECs, compared to β3WT ECs, as was extension into lamellipodia (Fig 3C; for an example of quantification of the latter, see Fig EV1). Overall, the findings suggest that β3‐integrin limits the targeting of MTs to FAs.

**Figure EV2:**
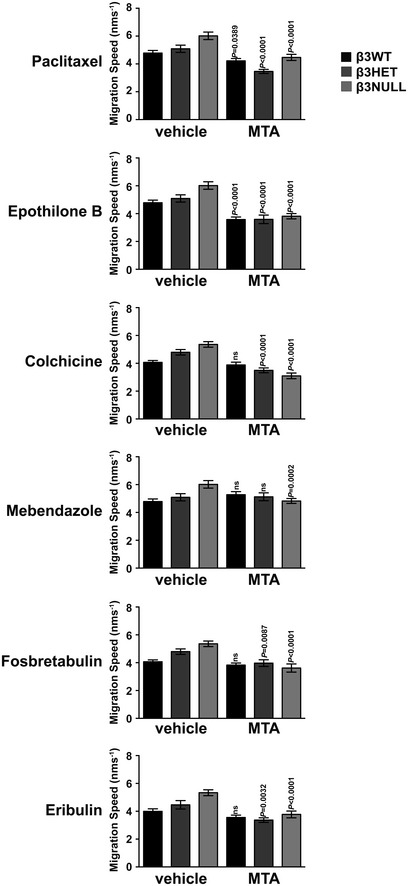
Effects of MTAs on EC migration speeds β3WT, β3HET and β3NULL endothelial cells were adhered to fibronectin overnight. Migration speed of individual cells was measured over 15 h using the MTrackJ plugin for ImageJ under the influence of the indicated MTA. Bars = mean migration speed (±SEM) (*n* ≥ 46 cells per genotype, from four independent experiments). Significant differences between means were evaluated by unpaired two‐tailed Student's *t‐*test.

Given that MTs can drive FA turnover and thus cell migration 18, we next tested whether EC migration is differentially sensitive to microtubule targeting agents (MTAs) in β3HET and β3NULL ECs. For each MTA examined, we first determined the dose of the compound that allowed 90% survival of β3WT ECs (see Materials and Methods) and then tested the effects of this dose on random migration in β3WT, β3HET and β3NULL cells (Fig 3D; raw migration data shown in Fig EV2). Random migration was affected by MT stabilisers (Paclitaxel, Epothilone B) in cells of all three genotypes, However, β3WT ECs were insensitive to the MT destabilisers tested (Colchicine, Mebendazole, Fosbretabulin) and the mechanistically unique MTA Eribulin (which functions through an end poisoning mechanism 19), whilst β3HET and β3NULL ECs generally showed a sensitivity to all classes of compounds tested. We extended these types of analyses *in vivo* to examine the effects of Eribulin and Fosbretabulin on tumour growth and angiogenesis. We chose these two MTAs as they are well tolerated in mice 20, 21 and used clinically in humans. We settled on suboptimal doses (see Materials and Methods) that would allow us to observe potential synergy with endothelial depletion of β3‐integrin. β3‐integrin‐floxed/floxed mice 22 were bred with Tie1Cre mice 23 to generate β3‐integrin‐floxed/floxed Cre‐positive animals (Cre‐negative littermates were used as controls). CMT19T lung carcinoma cells were injected subcutaneously and allowed to establish for 7 days, at which point the MTAs were administered (see Materials and Methods for dosing regimes). Neither Eribulin nor Fosbretabulin had any effect on tumour growth in Cre‐negative animals compared with vehicle‐treated animals, but tumour growth was reduced in MTA‐treated Cre‐positive animals (Fig 3E). Analysis of tumours by immunostaining for blood vessels showed a reduction in intratumoral microvascular density only in sections from MTA‐treated Cre‐positive animals (Fig 3F). These studies suggested that the loss of endothelial β3‐integrin sensitises angiogenic responses to MT destabilisers both *in vitro* and *in vivo*.

**Figure 4:**
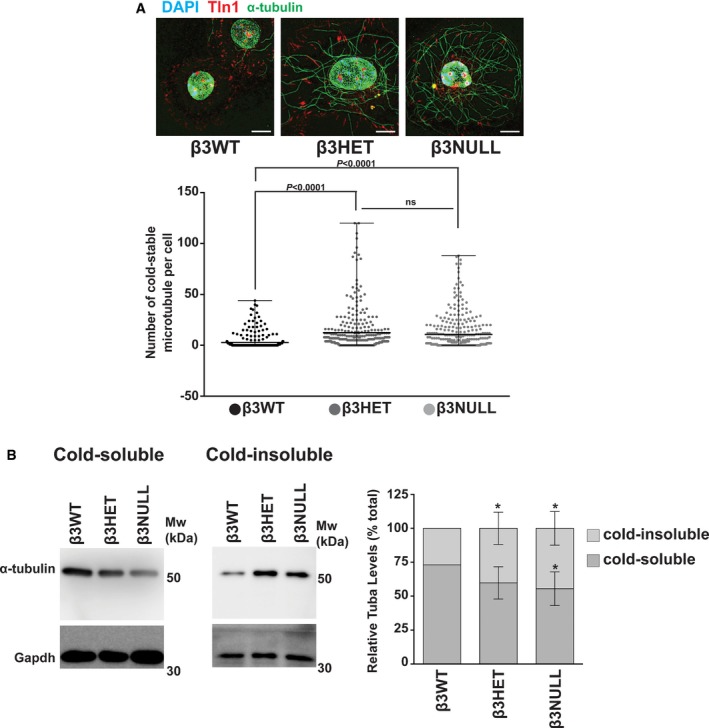
Analysis of microtubule stability in β3WT, β3HET and β3NULL endothelial cells *Top:* β3WT, β3HET and β3NULL endothelial cells were adhered to fibronectin‐coated coverslips for 75 min at 37°C before being moved to ice for 15 min. Soluble tubulin was then washed out using PEM buffer (see Materials and Methods) before fixing with −20°C methanol (Note: this protocol leads to nuclear auto‐fluorescent background in all three channels used). Immunostaining was carried out for α‐tubulin (green) and talin‐1 (Tln1, red). DAPI (blue) was used as a nuclear stain. Images shown are representative of the data shown in the bar graph shown below. Scale bar = 5 μm. *Bottom:* Dot plots = mean (±SEM) number of cold‐stable microtubules per cell (*n* ≥ 300 cells per genotype, from three independent experiments). Significant differences between means were evaluated by unpaired two‐tailed Student's *t‐*test.β3WT, β3HET and β3NULL endothelial cells were adhered to fibronectin for 75 min at 37°C before being moved to ice for 15 min. Cold‐soluble tubulin (*left blot*) was then washed out using PEM buffer and Western‐blotted for α‐tubulin and Gapdh (as a loading control). Cold‐insoluble tubulin (*middle blot*) from the same cells was obtained by lysing the remaining cells and Western blotting for α‐tubulin and Gapdh (as a loading control). *Right bar chart:* Bars = mean (±SEM) relative cold‐soluble and cold‐insoluble α‐tubulin levels for each genotype. Data are representative of four independent experiments. *indicates statistical significance compared to WT (*P* < 0.05). Significant differences between means were evaluated by unpaired two‐tailed Student's *t‐*test.

**Figure EV3:**
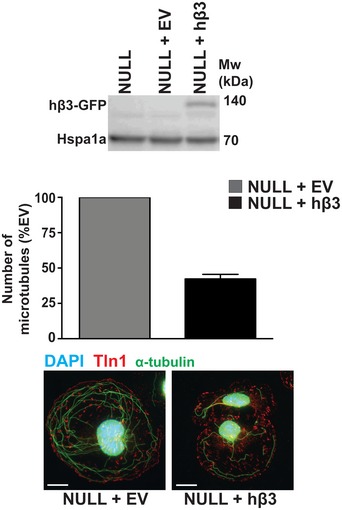
Measuring the effects on microtubule stability of reintroducing β3‐integrin into β3NULL ECs *Top:* β3NULL endothelial cells were transfected with a full‐length human β3‐integrin (hβ3) cDNA expression construct or an empty vector (EV) control and Western‐blotted for β3‐integrin (β3NULL parent cells shown for comparison). *Bottom:* β3NULL + EV or β3NULL + hβ3 endothelial cells were adhered to fibronectin‐coated coverslips for 75 min at 37°C before being moved to ice for 15 min. Soluble tubulin was then washed out using PEM buffer (see Materials and Methods) before fixing with −20°C methanol. Immunostaining was carried out for α‐tubulin (green) and talin‐1 (Tln1‐red). DAPI (blue) was used as a nuclear stain. Images shown are representative of the data shown in the bar graph above. Bars = mean (±SEM) number of cold‐stable microtubules per cell (*n* = 96 cells per genotype). Scale bar = 5 μm.

The increased sensitivity to destabilising MTAs suggested to us that there is an increased population of stable MTs in β3HET and β3NULL ECs compared with their wild‐type counterparts. We explored this premise by exposing ECs to cold temperatures (which destabilises MTs), washing out tubulin monomers 24, followed by immunolabelling for α‐tubulin. We noted elevated stable MTs in both β3HET and β3NULL cells (Fig 4A). Re‐introducing β3‐integrin into β3NULL cells restored MT sensitivity to cold; MTs were more sensitive to cold treatment in NULL cells expressing full‐length human β3‐integrin, than MTs in cells transfected with an empty vector control (Fig EV3). We also measured MT stability biochemically by extracting both cold‐sensitive and cold‐stable MTs from the same sample of cold‐treated cells and Western blotting for α‐tubulin (Fig 4B). On whole, β3HET and β3NULL ECs showed decreased cold‐sensitive and increased cold‐stable MTs compared with β3WT ECs.

**Figure 5:**
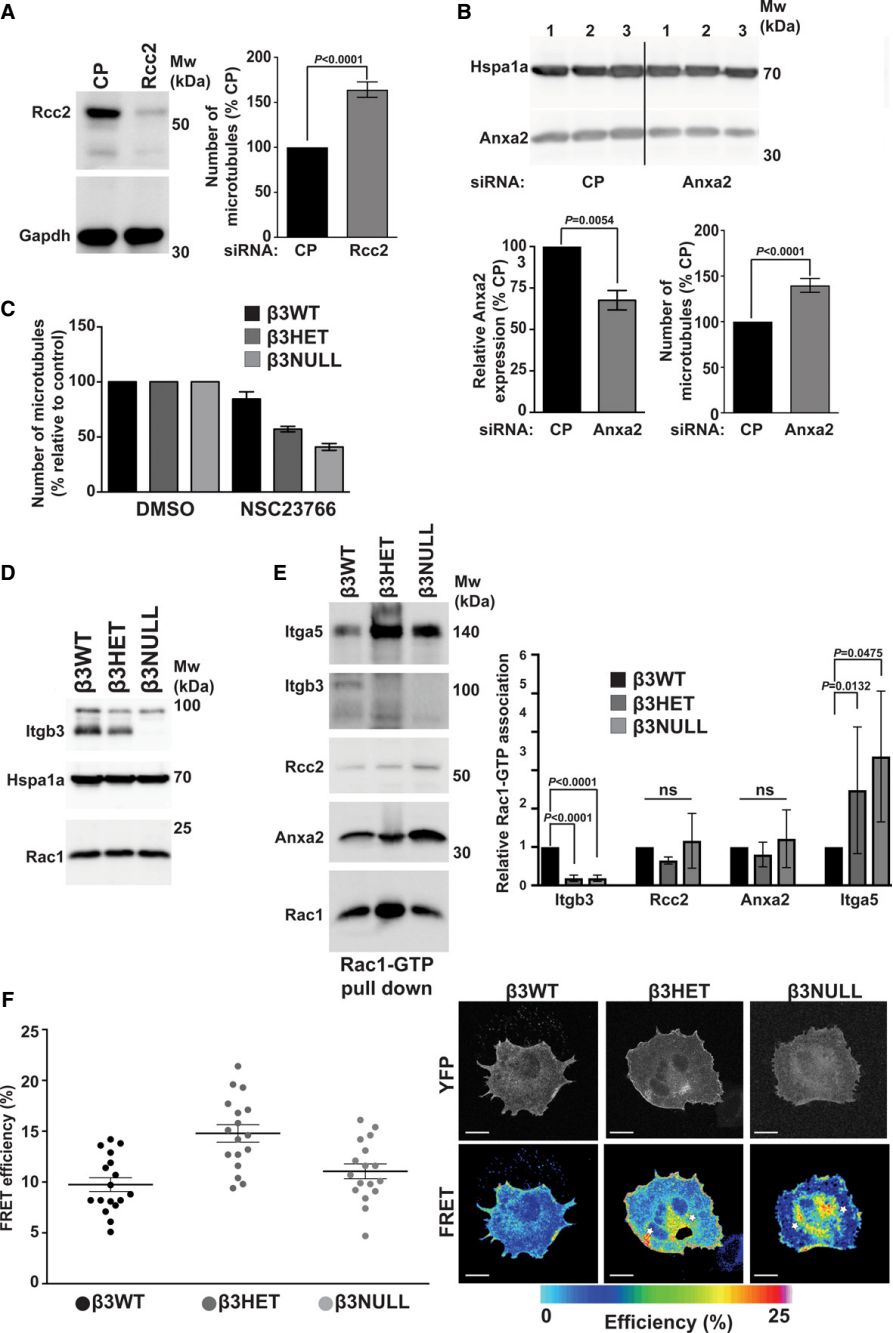
Microtubule stability in endothelial cells is regulated by Itgb3, Rcc2, Anxa2 and Rac1 β3WT ECs were transfected with control pool (CP) or Rcc2 smart pool siRNA and allowed to recover for 48 h. They were then adhered to fibronectin‐coated coverslips for 75 min at 37°C before being moved to ice for 15 min. Soluble tubulin was then washed out using PEM buffer before fixing with −20°C methanol. Immunostaining was carried out for α‐tubulin to allow counting of the number of cold‐stable microtubules per cell. *Left:* Western blot showing representative Rcc2 knockdown. Gapdh is shown as a loading control. *Right:* Bars = mean (±SEM) number of cold‐stable microtubules shown as a percentage relative to CP‐treated cells (*n* ≥ 455 cells per condition, from three independent experiments). Significant differences between means were evaluated by unpaired two‐tailed Student's *t‐*test.β3WT ECs were transfected with control pool (CP) or Anxa2 smart pool siRNA and allowed to recover for 48 h. They were then adhered to fibronectin‐coated coverslips for 75 min at 37°C before being moved to ice for 15 min. Soluble tubulin was then washed out using PEM buffer before fixing with −20°C methanol. Immunostaining was carried out for α‐tubulin to allow counting of the number of cold‐stable microtubules per cell. *Top:* Western blot showing representative Anxa2 knockdown in three separate samples. *Bottom left:* Bars = mean (±SEM) Anxa2 knockdown shown as a percentage relative to CP‐treated cells. Samples have been normalised to Hspa1a. *Bottom right:* Bars = mean (±SEM) number of cold‐stable microtubules shown as a percentage relative to CP‐treated cells (*n* ≥ 450 cells per condition, from three independent experiments). Significant differences between means were evaluated by unpaired two‐tailed Student's *t‐*test. The graph is representative of 3 independent experiments.β3WT, β3HET and β3NULL endothelial cells were adhered to fibronectin‐coated coverslips for 60 min at 37°C before being treated with DMSO (control) or 50 μM NSC23766 and incubated at 37°C for a further 15 min. Coverslips were moved to ice for 15 min. Soluble tubulin was then washed out using PEM buffer before fixing with −20°C methanol. Immunostaining was carried out for alpha‐tubulin to allow counting of the number of cold‐stable microtubules per cell. Bars = mean (±SEM) number of microtubules per cell shown as a percentage relative to DMSO‐treated controls (*n* = 218 cells per condition, from two independent experiments).β3WT, β3HET and β3NULL endothelial cells were adhered to fibronectin for 90 min before being lysed and Western‐blotted for integrin‐β3 (Itgb3), Rac1 and Hspa1a (as a loading control). Blot shown is representative of three individual experiments.β3WT, β3HET and β3NULL endothelial cells were adhered to fibronectin‐coated plates for 90 min before being lysed in MLB (see Materials and Methods). GTP‐Rac1 and bound proteins were extracted from cleared MLB using PAK‐1 PBD magnetic beads at 4°C for an hour before being Western‐blotted for Itga5, Itgb3, Rcc2, Anxa2 and Rac1. Blot is representative of at least three independent experiments *Right:* Bars = mean (±SD) level of association of the indicated protein with GTP‐Rac1, shown relative to β3WT associations (and normalised to the level of active Rac1 pulled down). Results are from at least three independent experiments. Significant differences between means were evaluated by unpaired two‐tailed Student's *t‐*test.β3WT, β3HET and β3NULL endothelial cells were transfected with a Raichu‐Rac1 biosensor. After 48 h, cells were adhered to fibronectin‐coated plates for 90 min and then fixed in PFA. *Left:* FRET efficiency was measured as described in Materials and Methods. Graph shows mean FRET efficiencies (±SEM; *n* = 17 cells per genotype, from two independent experiments). *Right:* Representative images showing spatial distribution of Rac1 FRET efficiency in β3WT, β3HET and β3NULL endothelial cells (white stars indicate cytoplasmic localisation of active Rac1 in β3HET and β3NULL). Scale bar = 5 μm.

**Figure EV4:**
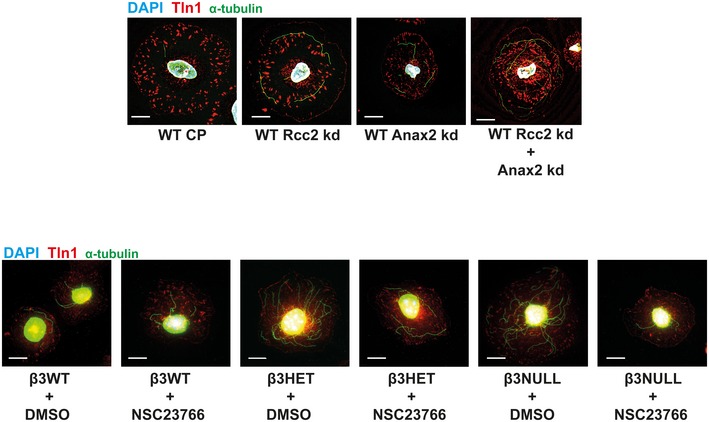
Representative microtubule staining in siRNA‐treated and NSC23766‐treated ECs *Top:* β3WT ECs were transfected with control pool (CP), Anxa2 smart pool siRNA, Rcc2 smart pool siRNA or both, and allowed to recover for 48 h. They were then adhered to fibronectin‐coated coverslips for 75 min at 37°C before being moved to ice for 15 min. Soluble tubulin was then washed out using PEM buffer before fixing with −20°C methanol. Immunostaining was carried out for α‐tubulin (green) and talin‐1 (Tln1‐red). DAPI (blue) was used as a nuclear stain. Scale bar = 5 μm. *Bottom:* β3WT, β3HET and β3NULL cells were adhered to fibronectin‐coated coverslips for 60 min at 37°C before treated with DMSO or 50 μM NSC23766 and incubated at 37°C for a further 15 min. Cells were then moved to ice for 15 min. Soluble tubulin was washed out using PEM buffer before fixing with −20°C methanol. Immunostaining was carried out for α‐tubulin (green) and talin‐1 (Tln1‐red). DAPI (blue) was used as a nuclear stain. Scale bar = 5 μm.

To gain further mechanistic insight into how β3‐integrin at FAs might be regulating MT function, we delved deeper into our β3‐dependent adhesome data. We noted that Rcc2 clusters with β3‐integrin in the β3WT adhesome, but is significantly decreased in that of β3HET ECs. Rcc2 (also known as telophase disc protein of 60 kDa, TD‐60) has previously been shown to associate with integrin complexes 25 and to regulate MTs 26. We therefore examined whether Rcc2 was regulating MT stability in ECs. Knocking down Rcc2 by siRNA in β3WT ECs elicited a significant increase in cold‐stable MTs (Fig 5A; see Fig EV4 for representative MT staining). This finding suggested to us that Rcc2 plays a β3‐dependent role in regulating MTs in ECs, but does not do so in isolation. We therefore cross‐referenced our adhesome data with an Rcc2 pull‐down assay performed from HEK‐293T cells (Table EV3) 27. Some obvious potential candidates (e.g. Coronin‐1C) were present in both the β3WT and β3HET adhesomes, but at the same level, so were ruled out from further analysis. However, annexin‐a2 (Anxa2) co‐precipitates with Rcc2 in HEK‐293T cells and, like Rcc2, was reduced in the β3HET adhesome. Therefore, we examined whether Anxa2 was also regulating MT stability in ECs via siRNA‐mediated knockdown. Like Rcc2 knockdown, even a relatively small (~ 30%) Anxa2 knockdown in β3WT ECs elicited a significant increase in cold‐stable MTs (Fig 5B; see Fig EV4 for representative MT staining). Moreover, a double knockdown of both targets led to an additive increase in cold‐stable MTs in β3WT ECs (Fig EV4).

**Figure EV5:**
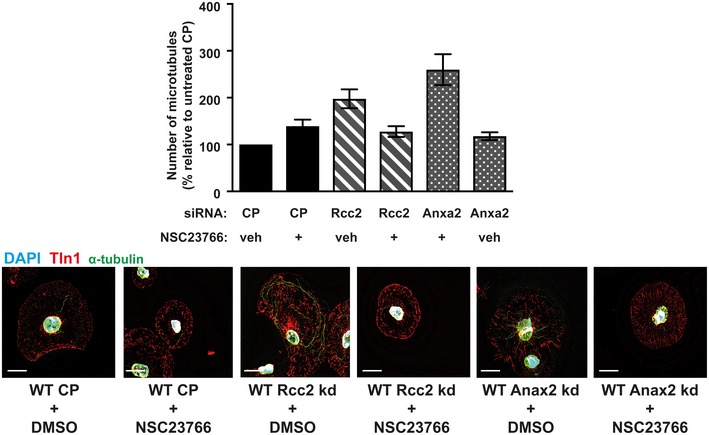
Measuring the effects on microtubule stability after NSC23766 administration in Rcc2 or Anax2 siRNA‐treated ECs *Top:* β3WT endothelial cells were transfected with control pool (CP), Anxa2 or Rcc2 smart pool siRNA and allowed to recover for 48 h. Cells were then adhered to fibronectin‐coated coverslips for 60 min at 37°C before treated with DMSO (veh) or 50 μM NSC23766 (+) and incubated at 37°C for a further 15 min. Coverslips were moved to ice for 15 min. Soluble tubulin was then washed out using PEM buffer before fixing with −20°C methanol. Immunostaining was carried out for alpha‐tubulin to allow counting of the number of cold‐stable microtubules per cell. Bars = mean (±SEM) number of microtubules per cell shown as a percentage relative to the CP/veh control (*n* = 100 cells per condition, from two independent experiments). Scale bar = 5 μm.

Both Rcc2 25, 27 and Anxa2 28 have been identified as regulators of Rac1, and work by a number of groups has demonstrated that cortical Rac1 activity promotes MT stability 29, 30, 31. Because total Rac1 stoichiometry was unchanged when comparing β3WT and β3HET EC adhesomes, we hypothesised that Rcc2/Anxa2‐dependent alterations in Rac1 activity were responsible for altered MT stability in β3HET and β3NULL ECs. First, we tested the premise that Rac1 plays a differential role in regulating MT stability in β3WT and β3‐depleted ECs by testing the effects of the Rac1 inhibitor NSC23766. NSC23766 had no effect on MT stability in β3WT cells, but the number of cold‐stable MTs in both β3HET and β3NULL ECs was reduced in the presence of the inhibitor (Fig 5C; see Fig EV4 for representative MT staining). We also demonstrated that the increases observed in MT stability upon Rcc2 or Anxa2 knockdown were abrogated in the presence of NSC23766 (Fig EV5), suggesting that both proteins regulate MT stability in ECs in a Rac1‐dependent manner.

Rcc2 has previously been reported to limit the activation of both Rac1 and Arf6 25. Indeed, Rcc2 can guide mesenchymal cell migration by trafficking Rac1 and controlling its exposure to GEFs 27. We therefore tested whether there were differences in Rcc2/Anxa2/active‐Rac1 associations between β3WT and β3‐depleted ECs. First, we examined total cellular levels or Rac1 and showed they were equivalent in all three cell lines (Fig 5D). PAK‐PBD pull‐downs of GTP‐bound Rac1 showed co‐association of all three proteins in β3WT, β3HET and β3NULL ECs (Fig 5E), so we concluded that changes in Rac1 activity alone were not responsible for alterations in MT stability in β3‐depleted cells. Humphries *et al* 25 showed that Rcc2 is recruited to α5β1‐FN complexes but not α4β1‐Vcam1 (vascular cell adhesion molecule‐1) complexes in cells expressing both α4‐ and α5‐integrins. Thus, we also tested associations between Rcc2, Anxa2 and α5‐integrin in β3WT and β3‐depleted ECs by PAK‐PBD pull‐downs. Rcc2, Anxa2, β3‐integrin and α5‐integrin were pulled down with Rac1‐GTP in β3WT ECs. Rcc2 and Anxa2 were also pulled down with Rac1‐GTP in β3HET and β3NULL ECS, whilst β3‐integrin‐Rac1‐GTP associations were lost and α5‐integrin‐Rac1‐GTP associations were increased (Fig 5E). Given the stoichiometry of α5‐integrin in the β3‐depleted adhesome is unchanged compared to the β3WT adhesome (Fig 2) whilst Rcc2 and Anxa2 levels are decreased, we speculated that a substantial proportion of the observed increase in Rcc2/Anxa2/active‐Rac1/Itga5 associations in β3‐depleted cells occur away from β3‐rich FAs, perhaps in recycling endosomes. Endocytic trafficking of Rac1 is required for the spatial restriction of signalling during mammalian cell migration 32. In support of this hypothesis, we demonstrated a redistribution of Rac1‐GTP in β3‐depleted ECs using a Raichu‐Rac1 biosensor (Fig 5F); compared to β3WT ECs, a substantial proportion of active Rac1 in β3HET and β3NULL cells appeared cytoplasmic. This redistribution of active Rac1 appeared to be independent of the total level of active Rac1 present in the cells; active Rac1 levels were only noticeably elevated in β3HET cells (Fig 5F).

By mining the FN‐β3‐integrin EC adhesome, not only we have generated a valuable tool for the integrin and angiogenesis communities, but also we have utilised the data to uncover a novel role for β3‐integrin in regulating MT function/stability during EC migration. We previously showed that endothelial Rac1 is only required for tumour growth and angiogenesis when β3‐integrin is absent 33, but the underlying mechanism for this observation has remained unclear. Our working hypothesis is that engagement of αvβ3‐integrin with FN at mature FAs localises an Rcc2/Anxa2/Rac1 containing complex to these sites, either preventing GTP‐Rac1 from participating in MT stability or actively destabilising MTs (our experiments do not allow us to distinguish between these two possibilities), perhaps by controlling its exposure to GEFs. When αvβ3 is not present, the complex associates with α5β1‐integrin instead, where it now has the opposite effect on MTs (see synopsis). This re‐positioning of Rac1 activity means that it plays a role in MT‐linked EC migration only when αvβ3 is not present in mature FAs. There is certainly precedence for β3‐integrin regulating spatial distribution of signalling pathway components in cells. For example, we previously showed that β3‐integrin plays a role in locally suppressing β1‐integrin in fibroblasts to promote persistent cell protrusion and migration by regulating interactions between vasodilator‐stimulated phosphoprotein (Vasp) and Rap1‐GTP‐interacting adaptor molecule (Apbb1ip/RIAM) 34. Moreover, MTs have recently been shown to target active β1‐integrins 35. Thus, it will be particularly pertinent to next determine the full composition of the Rcc2/Anxa2/Rac1‐GTP complex as many of the proteins that might be suspected to play a role in MT capture (e.g. Clip170 and Clasps) do not appear to be present in the EC adhesome 36; to gain a full picture of how MT stability/FA targeting is regulated in ECs, it will also be essential to establish how this complex behaves in α5β1‐deficient ECs.

Finally, it is worth considering how changes in levels of integrin expression might affect the cellular responses we have examined. Whilst, in general (e.g. sensitivity to MTAs, including cold), β3HET and β3NULL cells behaved similarly in the assays we employed, there are two notable differences: (i) β3NULL cells showed increased MT targeting to lamellipodia, compared to β3HET cells (Fig 3C), which might suggest altered MT dynamics between the two genotypes. It will be important to examine microtubule dynamics in greater detail (e.g. rates of growth, catastrophe and rescue) with changes in integrin expression patterns/levels. (ii) On fibronectin, β3HET cells showed increased Rac1‐GTP levels compared to β3NULL cells (Fig 5). This might relate to the increased total VEGFR2 levels noted in β3NULL 37 but not β3HET 17 cells. If VEGFR2 is playing a role here, we speculate it is separate from its known interactions with αvβ3‐integrin. VEGFR2 and αvβ3‐integrin interactions are augmented on vitronectin 38, and we do not detect VEGFR2 in our FN‐dependent EC adhesome (Fig 1). Notwithstanding, our findings suggest that once effective αvβ3‐integrin antagonists are available (e.g. ProAgio 39), they may be particularly useful as anti‐angiogenic agents when used in combination with already approved MTAs, such as Eribulin.

## Materials and Methods

### Reagents

Unless otherwise stated, all chemicals used were purchased from Sigma‐Aldrich (Poole, UK). Vascular endothelial growth factor (mouse VEGF‐A^164^) was made in house according to Krilleke *et al* 40.

### Animals

All animals were on a mixed C57BL6/129 background. Littermate controls were used for all *in vivo* experiments. All animal experiments were performed in accordance with UK Home Office regulations and the European Legal Framework for the Protection of Animals used for Scientific Purposes (European Directive 86/609/EEC).

### Mouse endothelial cell isolation and culture

Mouse lung ECs were isolated from adult mice on a mixed C57BL6/129 background as per Reynolds and Hodivala‐dilke 41 and then subsequently immortalised and cultured as per Ellison *et al* 17. The cell lines used in the studies presented here were cross‐referenced to a pure C57BL6 genetic background via a 384 single nucleotide polymorphism panel (Charles River Genetic Testing Services, Wilmington, MA, USA). These analyses showed the following: β3WT = 91.99% C57BL6; β3HET = 93.03% C57BL6; β3NULL = 44.75% C57BL6.

### Adhesion assay

Ninety‐six‐well plates were coated in 10 μg ml^−1^ fibronectin (FN) in phosphate buffered saline (PBS) overnight at 4°C and then blocked with 1% bovine serum albumin (BSA) in PBS for 1 h at room temperature. A total of 20,000 cells were seeded into each well and allowed to adhere for 90 min. Cells were then washed with PBS with 1 mM MgCl_2_ and 1 mM CaCl_2_ three times to remove non‐adherent cells and fixed with 4% paraformaldehyde (PFA) for 10 min at room temperature. After a further PBS wash, cells were stained with 1% methylene blue in 10 mM borate buffer pH 8.5/50% methanol for 30 min at room temperature. Excess stain was removed with RO water before a 50% 0.1 M HCl/50% ethanol destain solution was used for 10 min at room temperature. The destain solution was then moved to a new plate, and absorbance was measured at 630 nm.

### Focal adhesion enrichment

Focal adhesion enrichment was carried out as described in Ellison *et al* 17 and Schiller *et al* 13. A small amount of each focal adhesion sample generated was quality‐controlled by running a 10% SDS–PAGE gel followed by silver staining (Pierce™ Silver Stain Kit, Thermo Fisher Scientific, Cramlington, UK). Good‐quality samples were then analysed by Western blotting or mass spectrometry.

### Mass spectrometry (MS)

Mass spectrometry was carried out by the Fingerprints Proteomics Facility (Dundee University, Dundee, UK) as per Schiller *et al* 13. Peptides were identified and quantified using MaxQuant 42 software using the Andromeda peptide database. To achieve label‐free quantitative results, three biological repeats were pooled and each of these pooled samples was analysed via three technical repeats through the spectrometer.

### MS statistical analysis

All mass spec analysis was performed using the Perseus 43 bioinformatics toolbox for MaxQuant. Statistical significance was identified using the significance analysis of microarrays (SAM) method 44. Unsupervised hierarchical clustering was performed using Perseus’ built in tools. KEGG and GO annotations were obtained from the mouse annotations package via Perseus (downloaded 20/06/2015) and used to identify angiogenesis, cytoskeleton and focal adhesion‐related genes.

### Random migration

Twenty‐four‐well plates were coated with 10 μg ml^−1^ FN in PBS overnight at 4°C and then blocked with 1% BSA for 1 h at room temperature. A total of 10,000 ECs were seeded per well and allowed to recover overnight. Media were then replaced with media containing one of the following microtubule targeting agents (MTAs—from Abcam, Abingdon, UK, unless otherwise noted): Paclitaxel 5 nM, Epothilone B 1 nM, Colchicine 10 μM, Mebendazole 0.4 μM, Fosbretabulin 0.5 μM or Eribulin 1 μM (a kind gift from Katherine Weilbaecher, Washington University, MO, USA); DMSO was used as a vehicle control. A phase contrast image was taken of each well every 20 min using an inverted Axiovert (Zeiss) microscope for 15 h at 37°C and 5% CO_2_. The ImageJ plugin MTrackJ 45 was then used to manually track individual cells, and the speed of random migration was calculated.

### Microtubule stability assays

Microtubule cold stability assays were carried out as described in Ochoa *et al* 24. Briefly, 750,000 ECs were seeded per well of a six‐well plate (FN‐coated/BSA‐blocked as described earlier) and allowed to adhere for 75 min at 37°C before being moved to ice for 15 min. Cells were washed with PBS and then 100 μl of PEM buffer (80 μM PIPES pH 6.8, 1 mM EGTA, 1 mM MgCl_2_, 0.5% Triton X‐100 and 25% (w/v) glycerol) for 3 min. A second brief wash was performed with 50 μl PEM buffer. All PEM buffer was collected and pooled together with 150 μl EB buffer (3% SDS, 60 mM Sucrose, 65 mM Tris–HCL pH 6.8) at 2× concentration (representing cold‐soluble microtubules). Remaining material on the plate was then extracted using 300 μl of EB buffer (representing cold‐stable microtubules). Samples were then used in Western blotting analysis.

Additionally, the same procedure was used on ECs adhered to FN‐coated/BSA‐blocked coverslips (acid washed and bake‐sterilised before coating). They were treated as above except that after PEM washing, the slides were immediately immersed in −20°C 100% methanol for 20 min. Coverslips were then used in immunolabelling analysis.

### *In vivo* tumour growth assays

The syngeneic mouse lung carcinoma cell line (derived from C57BL6 mice) CMT19T was used to grow subcutaneous tumours in β3 fl/fl Tie1Cre‐positive (and Cre‐negative littermate control) mice. Under anaesthetic, mice were injected subcutaneously in the flank with 1 × 10^6^ cells. Tumours then grew for 7 days, at which point they were palpable through the skin, before the mice were treated with: (i) 0.15 mg kg^−1^ Eribulin (kindly provided by Katherine Weilbaecher, Washington, USA) intravenously once a week for 2 weeks; or (ii) 50 mg kg^−1^ Fosbretabulin intraperitoneally every 4 days. After 21 days, mice were culled and tumours were excised, photographed and measured for volume using a digital calliper. Tumours were bisected along the midline, fixed overnight in 4% paraformaldehyde, preserved for several days in cryoprotectant (20% sucrose, 2% poly(vinylpyrrolidone) in PBS), embedded in gelatine (8% gelatine, 20% sucrose, 2% poly(vinylpyrrolidone) in PBS) before being snap‐frozen and stored at −80°C.

### Focal adhesion and microtubule tracking

1 × 10^6^ ECs were transfected with a GFP‐tagged paxillin cDNA expression construct (provided by Maddy Parsons, KCL) by nucleofection. Cells were allowed to recover overnight before a fraction was seeded on FN‐coated/BSA‐blocked coverslips (acid washed and baked before coating) and adhered for 3 h. Cells were then treated with 100 nM SiRTubulin (Cytoskeleton Inc CY‐SC002) and 1 μM Verapamil overnight. Coverslips were imaged individually on an Axiovert (Zeiss) inverted microscope where one image of a GFP‐positive cell was taken every minute for 30 min at 37°C and 5% CO_2_ in green and far‐red channels. During imaging, media were replaced with Phenol red‐free OptiMEM® + 2% FBS containing 100 nM SiRTubulin and 1 μM Verapamil. The total area of adhesive fronts was assessed by measuring the growth of paxillin–GFP‐positive areas between the 1^st^ and 30^th^ image, and then, the number of microtubules that entered the adhesive front over 30 min was counted.

### Western blotting

For Western blot analysis of total tubulin levels, ECs were seeded at 750,000 per well of a FN‐coated/BSA‐blocked six‐well plate and allowed to adhere for 90 min before being lysed in EB buffer. For the microtubule stability assay and focal adhesion enrichment, samples were prepared as above. 20 μg from each sample was loaded onto 10% polyacrylamide gels and then transferred to a nitrocellulose membrane and incubated for 1 h in 5% milk powder in PBS with 0.1% Tween 20 (PBSTw), followed by overnight incubation in primary antibody diluted 1:1,000 in 5% BSA in PBSTw at 4°C. Primaries used were against integrin beta 3 (Cell Signalling 4702), alpha‐tubulin (Abcam 7291), Gapdh (Abcam 9484), Rcc2 (Abcam 70788), Hspa1a (clone B‐6 Santa Cruz Biotechnology), Anxa2 (Abcam 41803) and Itga5 (Cell Signalling 4705). The membranes were then incubated with the appropriate horseradish peroxidase (HRP)‐conjugated secondary antibody (Dako) diluted 1:2,000 in 5% milk in PBSTw for 1 h at room temperature. The blot was visualised using Piece® ECL Western Blotting Substrate Kit (Thermo Fisher) and chemiluminescence detected on a Fujifilm LAS‐3000 darkroom (Fujifilm UK Ltd, Bedford, UK).

### Immunolabelling cells

A total of 20,000 ECs were seeded onto FN‐coated/BSA‐blocked coverslips and adhered for 90 min before being washed with PBS and immersed in −20°C methanol for 20 min. Alternatively, cells were prepared as per the microtubule stability assay protocol described above. Coverslips were then washed with PBS, blocked for 10 min at room temperature with 0.5% BSA, 1% goat serum in PBS with 0.25% Triton X‐100 and incubated with primary antibody diluted 1:250 in PBS for 1 h at room temperature. After subsequent PBS washes, the coverslips were incubated with Alexa Fluor®‐conjugated secondary antibodies raised in donkey (Fisher Scientific) diluted 1:500 in PBS. Coverslips were washed again in PBS before being mounted onto slides using Prolong Gold® with DAPI (Fisher Scientific). Primaries used were anti‐alpha‐tubulin (Abcam 52866), anti‐paxillin (Abcam 32084) anti‐talin (Sigma T3287) and anti‐Nrp1 (R&D Systems AF566).

To quantify microtubule targeting focal adhesions, images were taken of stained cells using an epifluorescent microtubule, and then, the number of microtubules with an end overlapping with a focal adhesion was counted for each cell.

Simultaneous phalloidin (Thermo Fisher A12380) and alpha‐tubulin staining were carried out using PHEMO fixation 46.

### Immunolabelling tissue sections

Five‐micrometer cryosections were prepared from frozen tumours and stained as described previously 17. Primaries used were anti‐CD31 (R&D Systems AF3628, 1:500) and alpha‐smooth muscle actin (Abcam 5694; 1:1,000). Images were acquired on an Axioplan (Zeiss) epifluorescent microscope. Vessel density (immediately adjacent to, but not including, the tumour border) was measured by hand in three hot spots per section. The area of each counted region was calculated using ImageJ.

### siRNA knockdown

Knockdowns of Rcc2 and Anxa2 were achieved using 3 μg of Dharmacon ON‐TARGETplus SMARTpool siRNA (control smart pool used as knockdown control) per 1 × 10^6^ ECs in an Amaxa Nucleofector II (T‐005 setting). Cells were allowed to recover for 48 h to allow knockdown to take effect.

### Generation of human β3 integrin expressing cells

1 × 10^6^ β3NULL endothelial cells were transfected with 10 μg of MfeI (New England Biolabs, Hitchin, UK) linearised full‐length human β3‐integrin (see Robinson *et al* 47) cloned into pcDNA™ 6.2/C‐EmGFP (see Amaxa nucleofection above). An empty vector (EV) was used as a control. Forty‐eight hours post‐transfection cells were selected with 10 μg ml^−1^ of blasticidin (Thermo Fisher). Cells surviving 2 weeks were analysed for β3‐integrin expression by Western blotting.

### Active Rac1 pull‐down

6 × 10^6^ ECs were seeded onto FN‐coated/BSA‐blocked (as described above) 10‐cm plates and allowed to adhere for 90 min. Rac1 Activation Magnetic Beads Pull‐down Assay Kit (Millipore 17‐10393) was then used per manufacturer's instructions. Pull‐down material was then loaded directly onto a gel for Western blotting.

### Rac1 biosensor analyses

1 × 10^6^ ECs were transfected with 10 μg of Raichu‐1011X (a gift from Maddy Parsons, KCL) via an Amaxa Nucleofector II (T‐005 setting). Cells were allowed to recover for 48 h and then plated onto FN‐coated/BSA‐blocked (as described above) coverslips for 90 min. Cells were fixed for 10 min in 4% PFA and then mounted in Prolong Gold® without DAPI (Thermo Fisher).

Samples for analysis of the Rac FRET biosensor by acceptor photobleaching were imaged and analysed as previously described 48. Briefly, images were acquired using an inverted Nikon A1R laser scanning confocal microscope. The CFP and YFP channels were excited using the 440‐nm diode laser and the 514‐nm argon line, respectively. The two emission channels were split using a 545‐nm dichroic mirror, which was followed by a 475‐ to 525‐nm bandpass filter for CFP and a 530‐nm longpass filter for YFP (Chroma). Pinholes were opened to give a depth of focus of 2 μm for each channel. Scanning was performed on a sequential line‐by‐line basis for each channel. The gain for each channel was set to approximately 75% of dynamic range (12‐bit, 4,096 grey levels), and offsets were set such that backgrounds were zero. Time‐lapse mode was used to collect one pre‐bleach image for each channel followed by bleaching with a minimum of 20 iterations of the 514‐nm argon laser line at maximum power (to bleach YFP). A second post‐bleach image was then collected for each channel. Control non‐bleached areas were acquired for all samples in the same field of view as bleached cells to confirm specificity of FRET detection. Pre‐ and post‐bleach TFP and Venus images were then imported into ImageJ for processing. Briefly, images were smoothed using a 3 × 3 box mean filter, background subtracted and post‐bleach images fade‐compensated. A FRET efficiency ratio map over the whole cell was calculated using the following formula: (TFP_post‐bleach_ − TFP_pre‐bleach_)/TFP_post‐bleach_. Ratio values were then extracted from pixels falling inside the bleach region as well as an equally sized region outside of the bleach region, and the mean ratio was determined for each region and is plotted on a histogram. The non‐bleach ratio was then subtracted from the bleach region ratio to give a final value for the FRET efficiency ratio. Data from images were used only if YFP bleaching efficiency was > 70%.

### Statistical analyses

All statistical tests were performed using GraphPad Prism™ Software. Significant differences between means were evaluated by unpaired two‐tailed Student's *t‐*test. *P* < 0.05 was considered statistically significant. Exact *P*‐values are shown in figures, except where *P* < 0.0001; ns = *P* > 0.05.

### Data availability

The mass spectrometry proteomics data have been deposited to the ProteomeXchange Consortium via the PRIDE 49 partner repository with the dataset identifier PXD008591. All additional data can be accessed by contacting the corresponding author.

## Author contributions

SJA designed and performed experiments, analysed data, and helped write and edit the manuscript. AMG, TSE, RTJ, BEH and MP designed and performed experiments, analysed data and helped edit the manuscript. BMK, AAAA, WJF and BCS performed experiments, analysed data and helped edit the manuscript. JGS, KNW and MDB provided essential data and helped edit the manuscript. MMM and DRE analysed data and helped edit the manuscript. SDR designed experiments, performed experiments, analysed data and wrote the manuscript.

## Conflict of interest

The authors declare that they have no conflict of interest.

## Supporting information



Expanded View Figures PDF

Table EV1

Table EV2

Table EV3

Review Process File
