# Supplementary material for: The β3‐integrin endothelial adhesome regulates microtubule‐dependent cell migration
Source: EMBO Rep. 2018 May 24;19(7):e44578. doi: 10.15252/embr.201744578 (PMC6030693; doi:10.15252/embr.201744578)
Supplement: Supplementary file 1 — Expanded View Figures PDF [file EMBR-19-e44578-s001.pdf]

Expanded View Figures

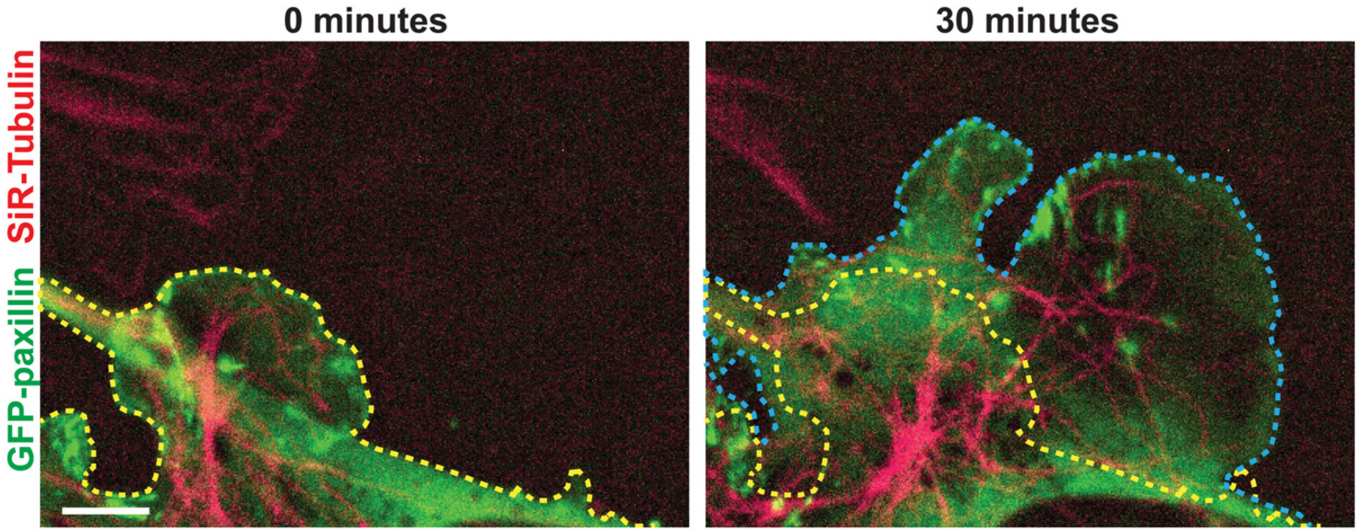

Figure EV1. Measuring microtubule extension into lamellipodium.

Schematic demonstrating how Fig 3C (right) was calculated. The yellow line indicates the edge of Pxn-GFP (green)-positive areas at 0 min, and the blue line indicates the edge at the end of 30 min. Microtubules were labelled red with SiRTubulin. Scale bar = 5 μm.

© 2018 The Authors

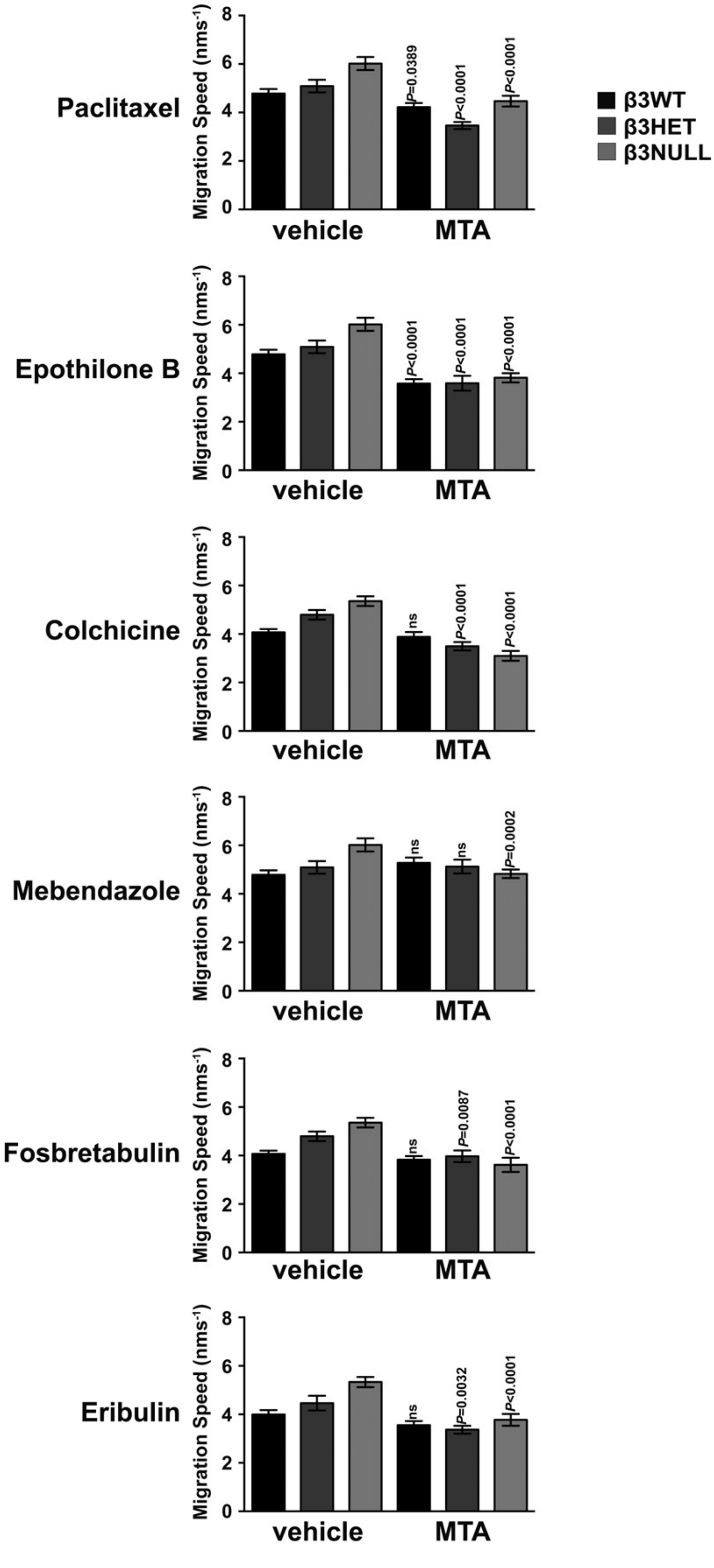

Figure EV2. Effects of MTAs on EC migration speeds.

β3WT, β3HET and β3NULL endothelial cells were adhered to fibronectin overnight. Migration speed of individual cells was measured over 15 h using the MTrackJ plugin for ImageJ under the influence of the indicated MTA. Bars = mean migration speed (±SEM) ($n \geq 46$ cells per genotype, from four independent experiments). Significant differences between means were evaluated by unpaired two-tailed Student's *t*-test.

 © 2018 The Authors

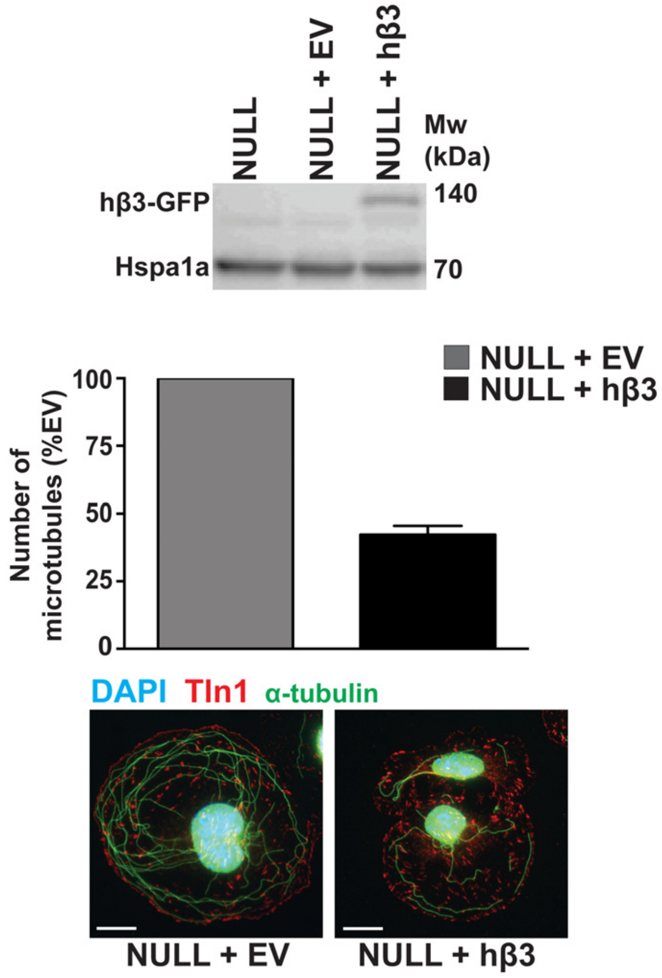

Figure EV3. Measuring the effects on microtubule stability of reintroducing β3-integrin into β3NULL ECs.

Top: β3NULL endothelial cells were transfected with a full-length human β3-integrin (hβ3) cDNA expression construct or an empty vector (EV) control and Western-blotted for β3-integrin (β3NULL parent cells shown for comparison). *Bottom:* β3NULL + EV or β3NULL + hβ3 endothelial cells were adhered to fibronectin-coated coverslips for 75 min at 37°C before being moved to ice for 15 min. Soluble tubulin was then washed out using PEM buffer (see Materials and Methods) before fixing with −20°C methanol. Immunostaining was carried out for α-tubulin (green) and talin-1 (Tln1-red). DAPI (blue) was used as a nuclear stain. Images shown are representative of the data shown in the bar graph above. Bars = mean (±SEM) number of cold-stable microtubules per cell (n = 96 cells per genotype). Scale bar = 5 μm.

© 2018 The Authors

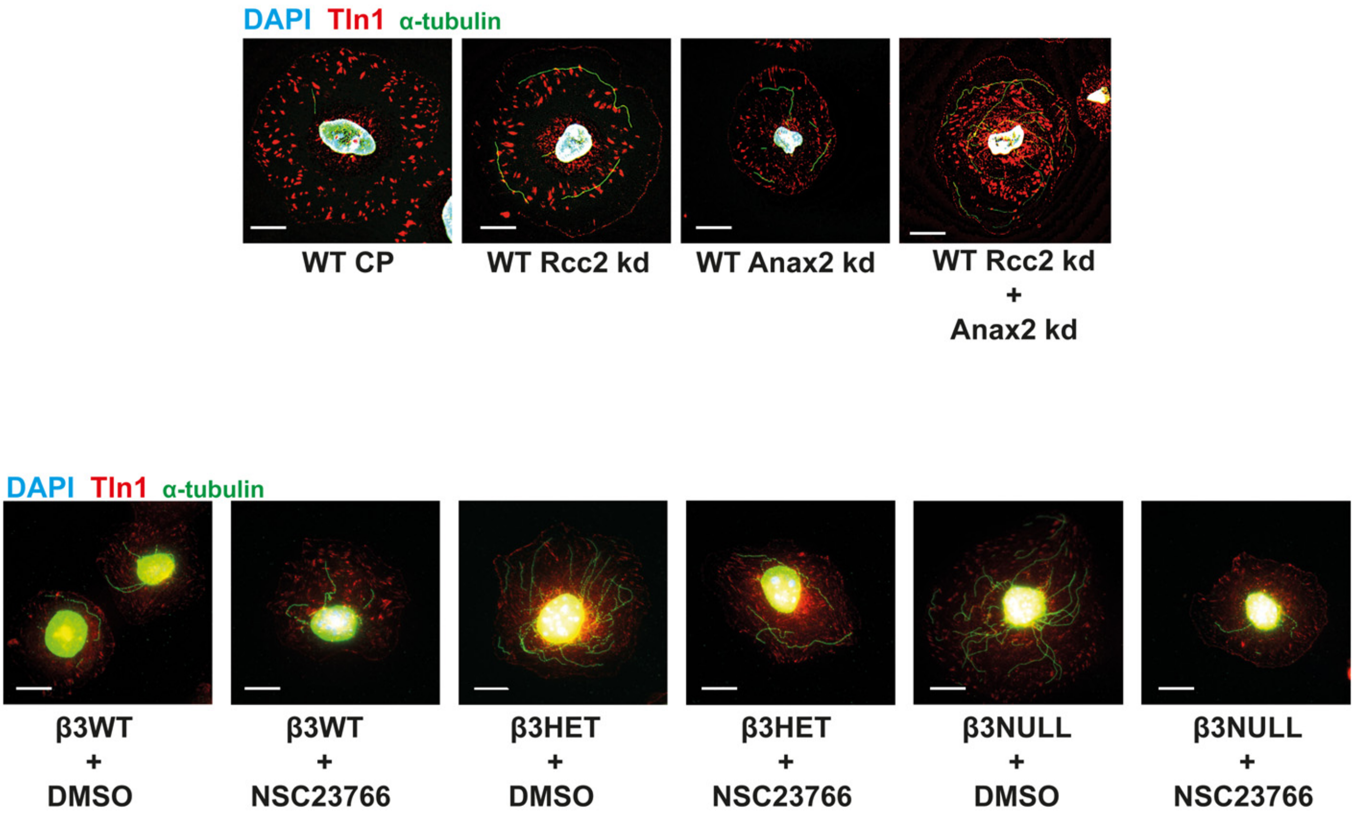

Figure EV4. Representative microtubule staining in siRNA-treated and NSC23766-treated ECs.

Top: β3WT ECs were transfected with control pool (CP), Anxa2 smart pool siRNA, Rcc2 smart pool siRNA or both, and allowed to recover for 48 h. They were then adhered to fibronectin-coated coverslips for 75 min at 37°C before being moved to ice for 15 min. Soluble tubulin was then washed out using PEM buffer before fixing with −20°C methanol. Immunostaining was carried out for α-tubulin (green) and talin-1 (Tln1-red). DAPI (blue) was used as a nuclear stain. Scale bar = 5 μm. *Bottom:* β3WT, β3HET and β3NULL cells were adhered to fibronectin-coated coverslips for 60 min at 37°C before treated with DMSO or 50 μM NSC23766 and incubated at 37°C for a further 15 min. Cells were then moved to ice for 15 min. Soluble tubulin was washed out using PEM buffer before fixing with −20°C methanol. Immunostaining was carried out for α-tubulin (green) and talin-1 (Tln1-red). DAPI (blue) was used as a nuclear stain. Scale bar = 5 μm.

© 2018 The Authors

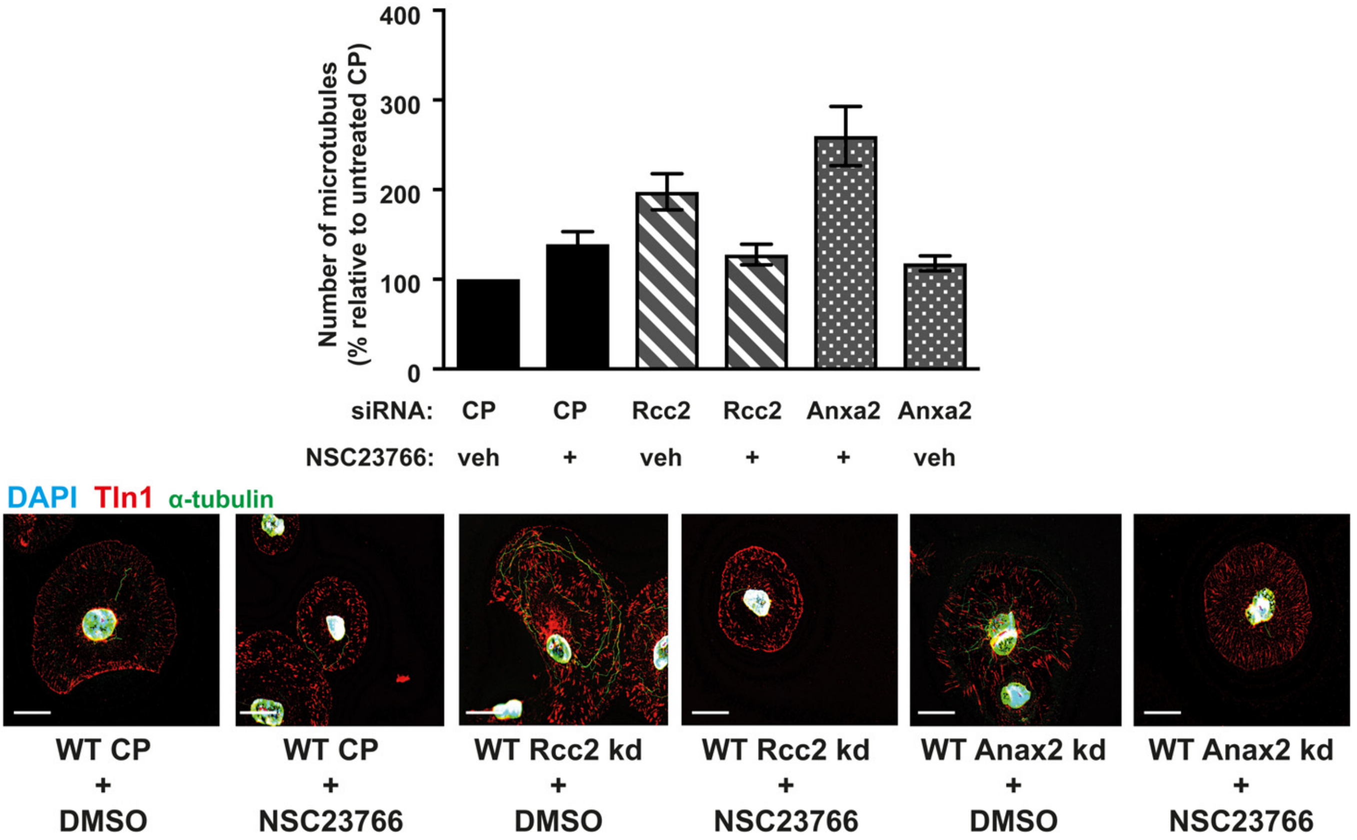

Figure EV5. Measuring the effects on microtubule stability after NSC23766 administration in Rcc2 or Anax2 siRNA-treated ECs.

Top: β3WT endothelial cells were transfected with control pool (CP), Anxa2 or Rcc2 smart pool siRNA and allowed to recover for 48 h. Cells were then adhered to fibronectin-coated coverslips for 60 min at 37°C before treated with DMSO (veh) or 50 μM NSC23766 (+) and incubated at 37°C for a further 15 min. Coverslips were moved to ice for 15 min. Soluble tubulin was then washed out using PEM buffer before fixing with −20°C methanol. Immunostaining was carried out for alpha-tubulin to allow counting of the number of cold-stable microtubules per cell. Bars = mean (±SEM) number of microtubules per cell shown as a percentage relative to the CP/veh control (*n* = 100 cells per condition, from two independent experiments). Scale bar = 5 μm.

© 2018 The Authors
