# Supplementary material for: The β3‐integrin endothelial adhesome regulates microtubule‐dependent cell migration
Source: EMBO Rep. 2018 May 24;19(7):e44578. doi: 10.15252/embr.201744578 (PMC6030693; doi:10.15252/embr.201744578)
Supplement: Supplementary file 5 — Review Process File [file EMBR-19-e44578-s005.pdf]

EMBO
reports

The β3-integrin endothelial adhesome regulates microtubule dependent cell migration

Samuel J Atkinson, Aleksander M Gontarczyk, Abdullah AA Alghamdi, Tim S Ellison, Robert T Johnson, Wesley J Fowler, Benjamin M Kirkup, Bernardo C Silva, Bronwen E Harry, Jochen G Schneider, Katherine N Weilbaecher, Mette M Mogensen, Mark D Bass, Maddy Parsons, Dylan R Edwards, Stephen D Robinson

Review timeline:

Submission date:	5 June 2017
Editorial Decision:	14 July 2017
Revision received:	23 December 2017
Editorial Decision:	8 March 2018
Revision received:	31 March 2018
Accepted:	27 April 2018

Editor: Martina Rembold

Transaction Report:

(Note: With the exception of the correction of typographical or spelling errors that could be a source of ambiguity, letters and reports are not edited. The original formatting of letters and referee reports may not be reflected in this compilation.)

1st Editorial Decision 14 July 2017

Thank you for the submission of your manuscript to our journal. We have now received the full set of referee reports that is copied below.

As you will see, while the referees agree that the study is potentially interesting, they also all point out that it requires significant revision before it can be considered for publication here. The major concerns regard missing quantification and control experiments, not fully conclusive evidence for the proposed Rac-Rcc2-Anxa2 pathway as well as several technical concerns.

From the referee comments it is clear that a major revision is necessary to substantiate the proposed concepts and to strengthen the data. On the other hand, given the potential interest of your findings and the constructive referee comments, I would like to give you the opportunity to address the concerns and would be willing to consider a revised manuscript with the understanding that the referee concerns must be fully addressed and their suggestions (as detailed above and in their reports) taken on board.

Should you decide to embark on such a revision, acceptance of the manuscript will depend on a positive outcome of a second round of review and I should also remind you that it is EMBO reports policy to allow a single round of revision only and that, therefore, acceptance or rejection of the manuscript will depend on the completeness of your responses included in the next, final version of the manuscript.

Revised manuscripts should be submitted within three months of a request for revision; they will otherwise be treated as new submissions. Please contact us if a 3-months time frame is not sufficient for the revisions so that we can discuss the revisions further.

Supplementary/additional data: The Expanded View format, which will be displayed in the main HTML of the paper in a collapsible format, has replaced the Supplementary information. You can

submit up to 5 images as Expanded View. Please follow the nomenclature Figure EV1, Figure EV2 etc. The figure legend for these should be included in the main manuscript document file in a section called Expanded View Figure Legends after the main Figure Legends section. Additional Supplementary material should be supplied as a single pdf labeled Appendix. The Appendix includes a table of content on the first page, all figures and their legends. Please follow the nomenclature Appendix Figure Sx throughout the text and also label the figures according to this nomenclature. For more details please refer to our guide to authors.

Regarding data quantification, please ensure the number of biological replicates (n), the bars and error bars (e.g. SEM, SD) and the test used to calculate p-values in the respective figure legends. Please also include scale bars in all microscopy images.

We now strongly encourage the publication of original source data with the aim of making primary data more accessible and transparent to the reader. The source data will be published in a separate source data file online along with the accepted manuscript and will be linked to the relevant figure. If you would like to use this opportunity, please submit the source data (for example scans of entire gels or blots, data points of graphs in an excel sheet, additional images, etc.) of your key experiments together with the revised manuscript. Please include size markers for scans of entire gels, label the scans with figure and panel number, and send one PDF file per figure or per figure panel.

When submitting your revised manuscript, we will require:

- a complete author checklist, which you can download from our author guidelines (http://embor.embopress.org/authorguide#revision). Please insert page numbers in the checklist to indicate where the requested information can be found.
- a letter detailing your responses to the referee comments in Word format (.doc)
- a Microsoft Word file (.doc) of the revised manuscript text
- editable TIFF or EPS-formatted figure files in high resolution
- a separate PDF file of any Supplementary information (in its final format)
- all corresponding authors are required to provide an ORCID ID for their name. Please find instructions on how to link your ORCID ID to your account in our manuscript tracking system in our Author guidelines (http://embor.embopress.org/authorguide).

We would also welcome the submission of cover suggestions, or motifs to be used by our Graphics Illustrator in designing a cover.

As part of the EMBO publication's Transparent Editorial Process, EMBO reports publishes online a Review Process File to accompany accepted manuscripts. This File will be published in conjunction with your paper and will include the referee reports, your point-by-point response and all pertinent correspondence relating to the manuscript.

You are able to opt out of this by letting the editorial office know (emboreports@embo.org). If you do opt out, the Review Process File link will point to the following statement: "No Review Process File is available with this article, as the authors have chosen not to make the review process public in this case."

I look forward to seeing a revised version of your manuscript when it is ready. Please let me know if you have questions or comments regarding the revision.

REFEREE REPORTS

Referee #1:

Stemming from the characterization of the β3 integrin adhesome, in their manuscript Atkinson and colleagues outline a potentially interesting model in which focal-adhesion-located αvβ3 would signal to destabilize microtubules, while α5β1, apparently when localized outside focal adhesion, would promote microtubule stabilization. Albeit novel and thought-provoking, the manuscript needs

to be significantly improved, several issues must be clarified, and additional experiments must be performed to formally or more thoroughly support concepts.

1. In general terms, it is not clear why Authors do not limit their studies to β3WT, β3HET, and β3NULL ECs. They do not need other cellular models. They should start comparing the three EC populations from the very beginning, rather than comparing β3WT and β3HET ECs first, and adding β3NULL ECs afterwards. Furthermore, data obtained from immortalized microvascular ECs isolated from C57BL6/129Sv mixed background mice and then treated or not with EMD66203 are quite problematic:

A. Page 3, first para. Due to the crucial role that the genetic background plays in influencing the cardiovascular phenotype (e.g. see George EL, Baldwin HS, Hynes RO. Fibronectins are essential for heart and blood vessel morphogenesis but are dispensable for initial specification of precursor cells. Blood. 1997; 90:3073-3081; Astrof S, Kirby A, Lindblad-Toh K, Daly M, Hynes RO. Heart development in fibronectin-null mice is governed by a genetic modifier on chromosome four. Mech Dev. 2007. 124:551-558.), it is important that Authors genetically define the exact percentage of the C57BL6/129Sv mixed background mice from which they isolated ECs.

B. Page 3, second para and Figure 1c. I do not see cluster B.

C. Page 4, second para and Figure 2a. Authors state: "EMD66203 inhibited EC adhesion to FN". Even if statistically significant, the percentage of inhibition of EC adhesion to FN is really minimal, so it not surprising that Authors found that "EMD66203... had no dramatic effect on the endothelial adhesome". I would suggest removing these data from the manuscript. It is not clear which is the message Authors would like to convey to the reader.

2. Page 5 second para and Figure 3a. Detailed zooms of pictures should be shown and the 'increased bundling' of microtubules must be precisely quantified.

3. Figure 3c. Which was the sample size (n)?

4. Page 5 third para and Figure 3d. Authors state "in general, β3NULL cells were more sensitive than β3HET cells". Was this difference statistically significant? This should be verified.

5. Figure 5f. $p = 0.08$ between Cre+/Veh and Cre+/Eri is not statistically significant, i.e. the positive control is lacking, and the panel should be removed. Which was the sample size (n)?

6. Page 7 and Figure 5a-d. Representative immunofluorescence pictures from which the microtubule graphs were generated must be shown.

7. Figure 5e. Total Rac1 must be shown, otherwise no conclusion can be drawn (e.g. the higher amount of Rac1-GTP, Anxa2, and α5 integrin in the β3HET lane may be due to higher content of total Rac1). The quality of the β3 western blot is quite poor. Again: which was the size (n) of samples plotted in the graph? I do not see any statistical analysis. Are the differences among samples statistically significant?

8. Page 7, last para. Authors state: "Rcc2... has been suggested that it functions as a Rac1 guanine nucleotide exchange factor (GEF)(Humphries et al., 2009)". This is not correct. Indeed, the hypothesis that Rcc2 might be a Rac1 GEF was formulated by Mollinari et al. (Dev. Cell. 2003. 5: 295-307), cited by Humphries et al., 2009. However, the conclusion of Humphries et al. 2009 was instead "that, rather than acting as a GEF, RCC2 limits activation of both Rac1 and Arf6".

9. To give more strength to their working model Authors should carefully analyze the subcellular localization of the different components of the Rcc2/Anxa2/Rac1 complex with respect to focal adhesions (vinculin, paxillin, and tensin), α5β1, and vesicular compartments (early and late endosomes, ER, and Golgi compartments) in β3WT, β3HET, and β3NULL ECs.

Referee #2:

© European Molecular Biology Organization

The β3-integrin endothelial adhesome regulates microtubule dependent cell migration

The authors characterized by mass spectrometry the molecular composition of the mature endothelial adhesome, and found that all detected tubulins were significantly upregulated in β3 heterozygous cells. They showed that depletion of β3-integrin leads to a reduction in the stability of microtubules through Rcc2, Anxa2, and Rac1 activity. This work proposes a novel role for β3-integrin in regulating microtubule stability in endothelial cells, and suggests that loss of β3-integrin expression might sensitize cells to microtubule targeting agents.

Specific comments:

1) The authors showed that depletion of β3-integrin in endothelial cells leads to an increase in microtubule stability. It would also be helpful to know whether antibody-mediated blockade of β3-integrin leads to similar effects on microtubule behavior.

2) Figure 3E-F shows that loss of β3-integrin expression in endothelial cells might provide beneficial sensitization of tumor growth to Eribulin treatment in vivo. However, it is unclear whether this effect is accompanied by a reduction in tumor angiogenesis. Although there is a trend, the data do not seem to be significant (p value is 0.08). To clarify,, the authors could quantify the vasculature using CD34/CD31 staining. At the same time, they should also test others drugs that destabilize microtubules (Colchicine, Mebendazole, Fosbretabulin) to confirm and strengthen their observation. Figure 3D shows that these drugs affect the migration speed to a similar extent than Eribulin.

3) The authors proposed that depletion of β3-integrin expression leads to an increased activity of Rac1, which favors microtubule stability. However, there is no data that show that Rac1 activity is increased in β3 heterozygous or null cells. Pull-down experiments or FRET imaging using the Raichu Probe are required to fill the gap. Western blot of total cell lysates should also be presented in Figure 3E. Surprisingly, depletion of β3-integrin did not lead to an increased activity of Rac1 in this particular case. It is unclear how the authors quantify the relative association of target proteins to Rac1-GTP. Did they normalize to activated Rac1? The data for Anxa2 are confusing. The Western blots show that Rac1 association with Rcc2 and Anxa2 is strongly increased in β3-depleted cells, definitely more than in wild-type and heterozygous cells, in contrast to the bar graphs.

4) Figure 3A is confusing. The average number of microtubules per cell seems low. Is there only one microtubule in wild-type cells? The micrographs would argue that this is indeed the case. Please comment.

5) Figure 5 shows that RNAi silencing of Rcc2 and Anxa2 leads to an increase in the stability of microtubules to the same extent. Although the knockdown of Rcc2 is obvious, the efficiency of Anxa2 depletion is weak. These observations raise the issue of specificity when using siRNA. Rescuing expression of Anxa2, and less critical for Rcc2, would strengthen their requirement in the signaling pathways.

Referee #3:

The authors utilize a mass spectrometry approach to characterize the lung microvascular endothelial cell adhesome in cells cultured on fibronectin ECMs. Results show that knockdown of B3-integrin changes the stability of microtubules by limiting the targeting of microtubules to focal adhesions. The authors also attempt to uncover the regulatory molecules responsible for controlling B3-integrin effects on microtubules. While this paper is potentially interesting to the readership of EMBO Reports, there were a number of experiments missing in figures that need to be added, and in doing so, could potentially enhance the authors conclusions. In particular, the Rac-Rcc2-Anxa2 series of experiments could be bolstered by adding Rac-active studies or rescue experiments to determine the finer details of what role these proteins play in the model system. The following are a list of major and minor concerns:

Major Concerns:

1. Figure 2:

- Fig. 2a: Why does EMD66203 inhibit cell adhesion by 40%, but B3-HET cells adhere equally well to FN as B3WT? Why is there a BSA bar in this graph?

- Shouldn't FA size be measured with the EMD66203?

- The author's conclusions (page 4) don't offer a clear interpretion of the EMD data. I don't understand why the EMD data is in the paper if the authors cannot understand their results.

- The authors need to stain cells for avB3 integrin in the EMD and in the B3HET cells to show it is reduced (again, the EMD data should be removed without this additional experiment, doing this would answer the question).

2. Figure 3:

- Page 5 in text: How is MT targeting peripheral FAs measured? What is the difference between MTs targeting lamellipodia vs. FAs? I don't see any description of how this was quantified in the methods.

- Fig. 3c: How do the authors explain that there are "no gross changes in MTs"? Why are there more MTs targeting FAs in the B3HET than in the B3 nulls?

- Fig. 3c: How is it that the B3Null is not different than the B3HET in MTs targeting lamellipodia. Looking at the significant change between B3WT and B3HET, it seems impossible that the Null is not different from the B3HET.

- Fig. 3d: Where is the migration data in the absence of drug? The authors should show the effects of DMSO on migration, rather than setting DMSO to 100% for each condition. If the cells are affected (and they should be) by reducing or eliminating avB3, then the data comparison shown should vary in the DMSO condition. This will inherently effect the outcomes of each of the MTAs shown relative to the condition of B3 (WT vs. HET vs. Null).

- Fig. 3e: I do not understand the purpose of the doxorubicin experiment. The author's rationale is stated to stem from a report that "targeting B3-integrin increases the efficiency of drug delivery to tumours (Wong et al, 2015). How do any of the drug regimens described in this manuscript target B3? They are MTA and DNA damaging drugs. The targeting of B3 has been done here using gene manipulation of B3, not drug targeting. Thus, the rationale provided for these experiments is extraneous.

- Fig. 3e: In both the Cre negative and Cre positive Doxorubicin experiments, the tumor volume is vastly reduced (<400mm3) compared to the Cre neg plus/minus Eribulin groups. In fact, the doxorubicin data are similar only to the Cre positive plus Eribulin group. These data suggest to me that Doxorubicin inhibits B3-dependent vascular infiltration of tumors similar to the combination of B3 knockout plus Eribulin. The authors state that "we observed no difference in tumor growth or vessel density when comparing Cre-positive to Cre-negative animals." The authors need to compare Doxorubicin to Eribulin.

3. Figure 4:

- Fig. 4a: How were microtubules quantified? The images shown do not match the quantified data for the B3HET and B3Null.

- Fig. 4b: Quantification of the western shows no difference between B3HET and WT. This result does not fit with the conclusion on page 6, that "B3HET and B3NULL ECs showed decreased cold-sensitvie .... MTs compared with B3WT." This sentence should be corrected to accurately depict the data.

4. Figure 5:

- Fig. 5b: Can the authors offer some explanation as to how a 30% knockdown of the Anxa2 protein results in the same outcome on MTs as does the 90% knockdown of Rcc2? The authors need to perform a simultaneous knockdown of Rcc2 and Anza2.

- Fig. 5c: How is it that the Rac inhibitor has no effect on the number of MTs in B3 WT cells? If these results are physiologically relevant, the experiment should work in WT cells. The finding that inhibiting Rac has no effect on MTs in B3WT cells suggests that Rac1 activity is not normally involved in regulating the number of MTs, which antagonizes the authors conclusions about mechanism.

- Fig. 5d: The authors need to conduct these knockdown experiments using a Rac activator or constitutively activated form of Rac in order to substantiate their conclusions regarding the role of Rac regulation via Rcc2 and Anxa2.

- Fig. 5e: Why is there no Itgb3 in the B3HET column? This result is confusing given the data shown in Figure 2e.

5. Materials and Methods:

- MT stability assay: It is not clear from the description of this assay how the "cold-soluble" versus "cold-insoluble" samples were separated from one another. Additionally, it would be helpful for the authors to use consistent terminology regarding the stability assay experiments and to mention these two groups in the materials and methods section. Also, in this section of the methods the authors mention "control cells" used for these stability experiments, but I did not see any data showing the results of these "control" experiments.

- GFP-paxillin is mentioned as being used for microtubule/focal adhesion tracking in the materials and methods, while in the paper only talin is mentioned.

Minor Concerns:

1. The adhesome is defined twice, once in the introduction and again in the first paragraph of results/discussion.

2. There are a number of bold-type words throughout the paper.

3. There are spacing errors between a number of sentences and the reference cited.

1st Revision - authors' response 23 December 2017

Response to the reviewers' comments

Referee #1

1. In general terms, it is not clear why Authors do not limit their studies to β3WT, β3HET, and β3NULL ECs. They do not need other cellular models.
Sincere apologies, but we do not understand this comment, as these are the only cellular models that we have included in the manuscript.

They should start comparing the three EC populations from the very beginning, rather than comparing β3WT and β3HET ECs first, and adding β3NULL ECs afterwards.
We have further clarified why only HET cells were used for label-free quantitative MS analyses, rather than KO cells (page 4, beginning line 121). However, we do feel that adding in NULL cells for subsequent analyses adds weight to our arguments about β3-integrin in the adhesome regulating microtubule stability and active Rac1 spatial distribution, particularly in the Raichu-Rac1 biosensor studies illustrated now in Figure 6.

© European Molecular Biology Organization

Furthermore, data obtained from immortalized microvascular ECs isolated from C57BL6/129Sv mixed background mice and then treated or not with EMD66203 are quite problematic:

A. Page 3, first para. Due to the crucial role that the genetic background plays in influencing the cardiovascular phenotype (e.g. see George EL, Baldwin HS, Hynes RO. Fibronectins are essential for heart and blood vessel morphogenesis but are dispensable for initial specification of precursor cells. Blood. 1997; 90:3073-3081; Astrof S, Kirby A, Lindblad-Toh K, Daly M, Hynes RO. Heart development in fibronectin-null mice is governed by a genetic modifier on chromosome four. Mech Dev. 2007. 124:551-558.), it is important that Authors genetically define the exact percentage of the C57BL6/129Sv mixed background mice from which they isolated ECs.
Thank you for the suggestion, and the Hynes's lab references. We have conducted SNP analyses of the cell lines used, and this is now referred to in the materials and methods section (page 9, beginning line 308). The WT and HET cells used in our studies are quite similar to one another, whilst the NULLs are less C57/BL6-like in comparison. However, we feel we can address your concerns further with the following:

- HET and NULL cells behave very similarly in our studies, in spite of their differences.
- WT cells behave more like HET/NULL cells with rcc2 and/or anxa2 knockdown (see Figs 5B and 5C).
- NULL cells return to a WT phenotype with the re-introduction of beta3 integrin (new data presented in Fig 6B).

B. Page 3, second para and Figure 1c. I do not see cluster B.
Apologies, this is a really small group and difficult to see on Figure 1C, nevertheless, a label has now been added (also for group J).

C. Page 4, second para and Figure 2a. Authors state: "EMD66203 inhibited EC adhesion to FN". Even if statistically significant, the percentage of inhibition of EC adhesion to FN is really minimal, so it not surprising that Authors found that "EMD66203... had no dramatic effect on the endothelial adhesome". I would suggest removing these data from the manuscript. It is not clear which is the message Authors would like to convey to the reader.
We have removed these data from the manuscript.

2. Page 5 second para and Figure 3a. Detailed zooms of pictures should be shown and the 'increased bundling' of microtubules must be precisely quantified.
This statement has now been removed from the manuscript; further consultation with our in-house microtubule expert (co-author Mogensen) suggested the original statement was an over interpretation.

3. Figure 3c. Which was the sample size (n)?
This information has now been added to the legend for Figure 3.

4. Page 5 third para and Figure 3d. Authors state "in general, β3NULL cells were more sensitive than β3HET cells". Was this difference statistically significant? This should be verified.
The ambiguity of this statement has been corrected in the revised version of the manuscript (page 5, beginning line 161).

5. Figure 5f. $p = 0.08$ between Cre+/Veh and Cre+/Eri is not statistically significant, i.e. the positive control is lacking, and the panel should be removed. Which was the sample size (n)?
As suggested by Reviewer 2, we have re-quantified microvascular density in tumour sections using anti-CD31 (rather than anti-Endomucin). New data for both Erubilin and Fosbretabulin are now shown in Figure 3 (panel F); n is now indicated in the figure legend.

6. Page 7 and Figure 5a-d. Representative immunofluorescence pictures from which the microtubule graphs were generated must be shown.
These are shown now in Figures 4A and 4B; to save creating overly busy figures, all other examples are shown in Figure EV3.

7. Figure 5e. Total Rac1 must be shown, otherwise no conclusion can be drawn (e.g. the higher amount of Rac1-GTP, Anxa2, and α5 integrin in the β3HET lane may be due to higher content of

total Rac1). The quality of the β3 western blot is quite poor. Again: which was the size (n) of samples plotted in the graph? I do not see any statistical analysis. Are the differences among samples statistically significant?
Total Rac1 levels for all three genotypes is now shown in Figure 6A. We agree that the quality of the Itgb3 western blot is not as beautiful as the others. This was the case in all of our attempts, and suggests to us that we are close to the lower limit of detection. However, given the quantification shown in the graph, we feel this illustrates the significant reduction of Rac1-GTP associated with β3-integrin in HET and NULL cells. The data presented in the graph has now been corrected (normalised) for levels of active Rac1 pulled down in each genotype. N is now listed in the legend to the figure and statistical information is now indicated on the graph.

8. Page 7, last para. Authors state: "Rcc2... has been suggested that it functions as a Rac1 guanine nucleotide exchange factor (GEF)(Humphries et al., 2009)". This is not correct. Indeed, the hypothesis that Rcc2 might be a Rac1 GEF was formulated by Mollinari et al. (Dev. Cell. 2003. 5: 295-307), cited by Humphries et al., 2009. However, the conclusion of Humphries et al. 2009 was instead "that, rather than acting as a GEF, RCC2 limits activation of both Rac1 and Arf6".
This has been corrected in the revised version of the manuscript (page 7, line 234).

9. To give more strength to their working model Authors should carefully analyze the subcellular localization of the different components of the Rcc2/Anxa2/Rac1 complex with respect to focal adhesions (vinculin, paxillin, and tensin), α5β1, and vesicular compartments (early and late endosomes, ER, and Golgi compartments) in β3WT, β3HET, and β3NULL ECs.
Thank you for this suggestion. We have made an attempt at co-localising these proteins via fluorescent immune-labelling. However, we have not been able to show significant differences in cells knocked down for Rcc2 or Anxa2 compared to control cells (in the era of siRNA this is something we do routinely to convince ourselves that observed staining is specific for the target protein). Whilst this might not be too surprising for Anxa2 as the knockdown in our hands is only ~40%, this should not be an issue for Rcc2 staining, where we achieve a very substantial depletion of the protein. As a consequence, we are uncomfortable publishing the data. We have, instead, taken a different approach to visualising active Rac1 cellular distribution via a Raichu-Rac1 FRET biosensor. These results are presented in Figure 6C.

Referee #2

1) The authors showed that depletion of β3-integrin in endothelial cells leads to an increase in microtubule stability. It would also be helpful to know whether antibody-mediated blockade of β3-integrin leads to similar effects on microtubule behavior.
Whilst we feel this might add useful information to our understanding of beta3-integrin's functions in the EC adhesome, because of the perceived ambiguity of the EMD66203 work, it has been removed from the manuscript. As such, we are now keeping the focus of this manuscript on changes that occur with β3-integrin expression.

2) Figure 3E-F shows that loss of β3-integrin expression in endothelial cells might provide beneficial sensitization of tumor growth to Eribulin treatment in vivo. However, it is unclear whether this effect is accompanied by a reduction in tumor angiogenesis. Although there is a trend, the data do not seem to be significant (p value is 0.08). To clarify,, the authors could quantify the vasculature using CD34/CD31 staining.
Thank you for the suggestion. These new data are now shown in Figure 3F.

At the same time, they should also test others drugs that destabilize microtubules (Colchicine, Mebendazole, Fosbretabulin) to confirm and strengthen their observation. Figure 3D shows that these drugs affect the migration speed to a similar extent than Eribulin.
Additional *in vivo* data with Fosbretabulin is now included in Figures 3E and 3F.

3) The authors proposed that depletion of β3-integrin expression leads to an increased activity of Rac1, which favors microtubule stability. However, there is no data that show that Rac1 activity is increased in β3 heterozygous or null cells. Pull-down experiments or FRET imaging using the Raichu Probe are required to fill the gap.

We are trying to show that rather than activity of Rac1, cellular distribution of active Rac1 is important. Thanks for the suggestion of the Raichu probe. These data are now included in the manuscript (Figure 6C), and help support our hypothesis.

Western blot of total cell lysates should also be presented in Figure 3E [author querry: do you mean Figure 5?]. Surprisingly, depletion of β3-integrin did not lead to an increased activity of Rac1 in this particular case. It is unclear how the authors quantify the relative association of target proteins to Rac1-GTP. Did they normalize to activated Rac1? The data for Anxa2 are confusing. The Western blots show that Rac1 association with Rcc2 and Anxa2 is strongly increased in β3-depleted cells, definitely more than in wild-type and heterozygous cells, in contrast to the bar graphs.
Again, thank you for the suggestion. The data presented in the Rac1-GTP pulldown experiments (now Figure 6B) have now been normalised against the amount of Rac1-GTP pulled down.

4) Figure 3A is confusing [author querry: do you mean Figure 4A?]. The average number of microtubules per cell seems low. Is there only one microtubule in wild-type cells? The micrographs would argue that this is indeed the case. Please comment.
We think this can be clarified by highlighting that in these studies we have stained for microtubules after cold treatment, which destabilises most of the microtubules in WT cells (in many cases there are none left); numbers of cold-stable microtubules are significantly higher in HET and NULL cells. We now show the range of microtubules in cells of each genotype after cold treatment as individual points on the graph in Figure 4A. To help make comparisons between genotypes/treatment conditions, however, all subsequent graphs of these measurements are shown as a percentage relative to "control" cells. However, examples of the staining are now shown for each condition in Figure EV3.

5) Figure 5 shows that RNAi silencing of Rcc2 and Anxa2 leads to an increase in the stability of microtubules to the same extent. Although the knockdown of Rcc2 is obvious, the efficiency of Anxa2 depletion is weak. These observations raise the issue of specificity when using siRNA. Rescuing expression of Anxa2, and less critical for Rcc2, would strengthen their requirement in the signaling pathways.
We have attempted to address this, but have been unsuccessful in our attempts to over express Rcc2 or Anxa2. We have shown, however, that a double knockdown of both proteins has an additive effect on microtubule stability (Figure 5C), as suggested both the other reviewers.

Referee #3

1. Figure 2:

- Fig. 2a: Why does EMD66203 inhibit cell adhesion by 40%, but B3-HET cells adhere equally well to FN as B3WT? Why is there a BSA bar in this graph?

- Shouldn't FA size be measured with the EMD66203?

- The author's conclusions (page 4) don't offer a clear interpretion of the EMD data. I don't understand why the EMD data is in the paper if the authors cannot understand their results.

- The authors need to stain cells for avB3 integrin in the EMD and in the B3HET cells to show it is reduced (again, the EMD data should be removed without this additional experiment, doing this would answer the question).

As mentioned above in comments to Referee #1, the EMD66203 data have been removed from the manuscript.

2. Figure 3:
- Page 5 in text: How is MT targeting peripheral FAs measured? What is the difference between MTs targeting lamellipodia vs. FAs? I don't see any description of how this was quantified in the methods.

© European Molecular Biology Organization

An example of how targeting lamellipodia was quantified is now shown in Figure EV1, and described better in the materials and methods (page 12, beginning line 405). Additionally, the FA targeting quantification is further clarified in the legend to Figure 3C (page 20, line 709).

- Fig. 3c: How do the authors explain that there are "no gross changes in MTs"? Why are there more MTs targeting FAs in the B3HET than in the B3 nulls?
As mentioned above, we have removed the statement about microtubule bundling being different between the different genotypes. This statement on page 4, lines 149-150 refers to overall appearance of the arrays of microtubules in the cells.

- Fig. 3c: How is it that the B3Null is not different than the B3HET in MTs targeting lamellipodia. Looking at the significant change between B3WT and B3HET, it seems impossible that the Null is not different from the B3HET.
We did not feel the difference between the HET and NULL was an important comparison to be made, as we were interested in WT compared to these two genotypes. However, we have now added this significance bar for the reviewer.

- Fig. 3d: Where is the migration data in the absence of drug? The authors should show the effects of DMSO on migration, rather than setting DMSO to 100% for each condition. If the cells are affected (and they should be) by reducing or eliminating avB3, then the data comparison shown should vary in the DMSO condition. This will inherently effect the outcomes of each of the MTAs shown relative to the condition of B3 (WT vs. HET vs. Null).
These raw data are now shown in Figure EV2.

- Fig. 3e: I do not understand the purpose of the doxorubicin experiment. The author's rationale is stated to stem from a report that "targeting B3-integrin increases the efficiency of drug delivery to tumours (Wong et al, 2015). How do any of the drug regimens described in this manuscript target B3? They are MTA and DNA damaging drugs. The targeting of B3 has been done here using gene manipulation of B3, not drug targeting. Thus, the rationale provided for these experiments is extraneous.

- Fig. 3e: In both the Cre negative and Cre positive Doxorubicin experiments, the tumor volume is vastly reduced (<400mm3) compared to the Cre neg plus/minus Eribulin groups. In fact, the doxorubicin data are similar only to the Cre positive plus Eribulin group. These data suggest to me that Doxorubicin inhibits B3-dependent vascular infiltration of tumors similar to the combination of B3 knockout plus Eribulin. The authors state that "we observed no difference in tumor growth or vessel density when comparing Cre-positive to Cre-negative animals." The authors need to compare Doxorubicin to Eribulin.
As suggested, we have removed the doxorubicin data from the manuscript.

3. Figure 4:

- Fig. 4a: How were microtubules quantified? The images shown do not match the quantified data for the B3HET and B3Null.
We now show the range of microtubules in cells of each genotype after cold treatment as individual points on the graph in Figure 4A. To help make comparisons between genotypes/treatment conditions, however, all subsequent graphs of these measurements are shown as a percentage relative to "control" cells. However, examples of the staining are now shown for each condition in Figure EV3.

- Fig. 4b: Quantification of the western shows no difference between B3HET and WT. This result does not fit with the conclusion on page 6, that "B3HET and B3NULL ECs showed decreased cold-sensitvie .... MTs compared with B3WT." This sentence should be corrected to accurately depict the data.
Apologies, we were trying to make reference to all the studies presented in Figure 4. The has been corrected to say "On whole. . . ." (page 6, line 194).

4. Figure 5:

- Fig. 5b: Can the authors offer some explanation as to how a 30% knockdown of the Anxa2 protein

results in the same outcome on MTs as does the 90% knockdown of Rcc2? The authors need to perform a simultaneous knockdown of Rcc2 and Anza2.
As suggested, this double knockdown experiment has been added to the manuscript (Figure 5C).

- Fig. 5c: How is it that the Rac inhibitor has no effect on the number of MTs in B3 WT cells? If these results are physiologically relevant, the experiment should work in WT cells. The finding that inhibiting Rac has no effect on MTs in B3WT cells suggests that Rac1 activity is not normally involved in regulating the number of MTs, which antagonizes the authors conclusions about mechanism.
Apologies, but we are not quite sure what the referee is getting at here. We are trying to say that a change in the spatial distribution of active Rac1 is what is influencing microtubule stability, etc. . . We fell that the data we present is consistent with the hypothesis that Rac1 is not playing a role in WT cells, thus inhibiting Rac1 activity in WT cells would not be expected to effect MTs.

- Fig. 5d: The authors need to conduct these knockdown experiments using a Rac activator or constitutively activated form of Rac in order to substantiate their conclusions regarding the role of Rac regulation via Rcc2 and Anxa2.
We have now conducted Raichu-Rac1 biosensor analyses (Figure 6C), and hope this helps to substantiate our conclusions.

- Fig. 5e: Why is there no Itgb3 in the B3HET column? This result is confusing given the data shown in Figure 2e.
We hypothesise that this is due to the levels of β3-integrin associated with Rac1-GTP in HET cells being below our limit of detection.

5. Materials and Methods:

- MT stability assay: It is not clear from the description of this assay how the "cold-soluble" versus "cold-insoluble" samples were separated from one another. Additionally, it would be helpful for the authors to use consistent terminology regarding the stability assay experiments and to mention these two groups in the materials and methods section. Also, in this section of the methods the authors mention "control cells" used for these stability experiments, but I did not see any data showing the results of these "control" experiments.
This has been clarified in the materials and methods (page 1, beginning line 370). There is no mention of control cells in this section.

- GFP-paxillin is mentioned as being used for microtubule/focal adhesion tracking in the materials and methods, while in the paper only talin is mentioned.
This was a typo that has been corrected to paxillin (page 11, line 396).

Minor Concerns:

1. The adhesome is defined twice, once in the introduction and again in the first paragraph of results/discussion.
This has been taken out of the Introduction.

2. There are a number of bold-type words throughout the paper.
These have been removed.

3. There are spacing errors between a number of sentences and the reference cited.
These have been corrected.

2nd Editorial Decision — 8 March 2018

Thank you for the submission of your revised manuscript to EMBO reports. I apologize for my delayed response, but as you will see from the reports that are copied below, the referee's opinion remained divided. I have meanwhile discussed the reports further with the referees and also within the editorial team.

As you will see, referee 1 supports publication of the manuscript in EMBO reports in its current form. However, referee 2 raises concerns about differences in Rac1 activity levels between beta3-integrin heterozygous (HET) and knockout (NULL) cells. This referee points out that Rac1-GTP levels are only increased in HET but not NULL cells. Also referee 3 is concerned about discrepancies between HET and NULL cells. Importantly, this referee points out that the proteomics experiment was performed only on heterozygous but not homozygous knockout cells and that the reasoning to do so, appears inappropriate. If the phenotype of NULL cells is too extreme to be used for the analysis of the adhesome, the inclusion of these cells for functional studies later in the manuscript appears not to be justified, in the opinion of this reviewer. I have discussed this extensively with the reviewers and also with the editorial team. Both, referee 1 and 2 support the inclusion of the data obtained in NULL cells, since these provide valuable information for the functional analysis of microtubule targeting. Moreover, both referees support publication in EMBO reports - given that the remaining questions as outlined in the referee reports are addressed.
We note that the best possible solution would be the inclusion of proteomics data obtained from NULL cells. Even if these cells display extreme changes, the information - in comparison to the HET cells - would be of value. We however realize that this will be a rather time-consuming experiment, in particular at this stage. Given that at least two referees support publication in EMBO reports, we would therefore like to give you the opportunity to address the remaining concerns.

In particular, it will be important to address these remaining concerns:
- Referee 2 points out that Rac1-GTP levels are only elevated in HET but not NULL cells. Please comment on this discrepancy and a possible involvement of VEGFR2 in the text.
- Please also provide a normalization of Rac1-GTP levels to total Rac1 levels in the same experiment.
- and comment on the potential endosomes in Fig. 6C

- Referee 3 points out that there is a difference in in MT targeting between HET and NULL cells in Fig. 3C. Please discuss this difference in the text in the most appropriate manner.
- Please also address the concerns regarding Figure 4 and EV3, regarding the differences in tubulin staining. Please provide new stainings, if required.
- Finally, please comment on the differences between cold-soluble and -insoluble tubulin.

Browsing through the manuscript myself, I noticed a few editorial things that we need before we can proceed with the acceptance of your study.

- You have submitted your manuscript as Scientific Report. This article type can only contain up to 5 figures. Since you currently have 6 figures, I suggest to change it to a full Article. In this case, the Results and Discussion section have to be separated.

- Statistics: I noticed that in some cased the number of individual experiments was 2 (n=2, e.g., Fig 3, 4, 5, and 6). Please note that in these cases the application of statistical tests is not appropriate due to the small sample size. Please review all figure panels and apply statistical test only to data that is obtained from at least 3 three independent experiments.

- The Author Checklist will be published alongside your manuscript. Therefore, please update the information in the section F- Data Accessibility.

- Please provide a running title of max. 40 characters incl. spaces on the first page of the manuscript.

- Please provide up to five keywords.

- Please update the reference list to match the EMBO reports style. The abbreviation 'et al' should be used if there are more than ten authors and the first ten author names should be listed. You can download the respective EndNot file from our Guide to Authors (https://drive.google.com/file/d/0BxFM9n2lEE5oOHM4d2xEbmpxN2c/view)

I look forward to seeing a revised form of your manuscript when it is ready.

REFEREE REPORTS

Referee #1:
(supports publication in EMBO reports without further revision)

Referee #2:

The authors have undertaken new experiments to address the comments from the original review. They have addressed the most significant issues.

Remaining issues: They propose the idea that depletion of β3-integrin expression leads to a change in cellular distribution of Rac1, rather than a change in its activity. The data presented in Fig. 6 indicate that Rac1 activity is only increased in β3HET and not β3NULL? In addition, the change in the distribution of active Rac1 seems to occur only in β3HET and not β3NULL (Fig. 6C). They should explain why the level of β3-integrin depletion leads to different results on Rac1 activity and localization in a non-intuitive manner. The authors should normalize the level of Rac1-GTP to the total cellular level of Rac1 within the same experiment to strengthen conclusion on Rac1 activity (Fig. 6B). The bar graph in Fig. 6B needs some work: the legend on the x-axis is missing, and there is no need to break the y-axis. The sentence on line 258: "Rac1 levels were only significantly elevated in β3HET cells (Fig. 6C)." should be changed to "Active Rac1 levels were only significantly elevated in β3HET cells (Fig. 6C)."

Referee #3:

The authors have made a number of revisions that address many of the concerns raised in the original round of review. However, there remain several concerns that were specifically not addressed at all, or not addressed in a comprehensive manner. Additionally, some of the new data do not offer convincing support of existing data, and raise additional questions. Given these concerns, I cannot recommend publication of this manuscript in EMBO Reports.

Remaining Major Concerns:

1. Experimental Rationale: The authors rationale for not using the B3NULL cells (line 121-124) suggests that it is inappropriate to use B3NULL cells for their experiments. Yet this rationale disappears after figure 2, when the authors decided: "To increase the power of our studies, we felt it appropriate to now also include B3NULL ECs in our analyses." Either the B3 NULLs should be used throughout the experiments, or not at all. Also, if B3 integrin is important for binding to fibronectin ECMs, why do the WT and HETs adhere equally to fibronectin (line 127-128)? Further, what integrin is used by the B3NULLs to adhere to fibronectin ECMs?

2. Experimental Rationale (continued): Figure 3C: The authors response to questions regarding the measured differences and statistical comparisons between B3HET and B3NULLs in figure 3C is concerning. They state that "We did not feel the difference between the HET and NULL was an important comparison to be made....." If this is true then all of B3NULL data should be removed from the manuscript. There is no point in including irrelevant data comparisons that even the authors feel is not important. This statement directly contradicts the authors statement highlighted above in major concern #1.

3. Continued errors in figure and text labeling: Line 151-152, the authors state that MT colocalization with talin-1 at peripheral FAs was greater..... Figure EV1 shows SiR-tubulin and GFP-paxillin, not talin-1.

4. Unconvincing new data: Figure 4 and EV3: The images shown in Figure 4A and Figure EV3, panel C, do not show the same outcomes from identical experiments, and are therefore unconvincing. In Figure 4A, there is clearly more tubulin the cells in the HET and NULL conditions compared to the WT. However, in Figure EV3, the exact same experimental groups display

microtubule labeling that looks nothing like the microtubules shown in Figure 4A and shows little visible difference between the 3 groups. This requires explanation. Additionally, in both figures, the tubulin staining looks extremely poor. Why does the majority of the tubulin staining appear to be diffuse labeling inside the nucleus?

5. Additional unresolved concerns: Figure 4C and D: the authors need to explain how isolation of cold-soluble versus cold-insoluble tubulin from the same cells can result in a significant increase in cold-insoluble tubulin (4D, B3HET), but an insignificant decrease in the cold-soluble tubulin (4C, B3HET). Cold-soluble plus cold-insoluble should equal total tubulin for each group. The result suggests possible problems in data acquisition or analysis.

2nd Revision - authors' response 31 March 2018

Note, to adhere to the format of a Scientific Report, Figures 5 and 6 of the previous version have been (re)combined, with some elements moved to Expanded View Figures to accommodate this merger.

- Referee 2 points out that Rac1-GTP levels are only elevated in HET but not NULL cells. Please comment on this discrepancy and a possible involvement of VEGFR2 in the text.

- **This has been included on page 9, lines 296-302.**

- Please also provide a normalization of Rac1-GTP levels to total Rac1 levels in the same experiment.

- **FRET normalisation to intensity levels across multiple cells is not relevant or possible in the Raichu studies presented in Figure 5 as each FRET value for each cell is relative to the intensity of the probe in that individual cell. However, total Rac1 levels are not changed based on biochemical analysis (see Fig 5D) and cells analysed for FRET were all expressing very similar levels of the probe (based on intensity).**

- and comment on the potential endosomes in Fig. 6C.

- **[Now Figure 5F]. This has been included (and referenced) in the discussion, pages 7-8, lines 254-259.**

- Referee 3 points out that there is a difference in in MT targeting between HET and NULL cells in Fig. 3C. Please discuss this difference in the text in the most appropriate manner.

- **This is included on pages 8-9, lines 292-296.**

- Please also address the concerns regarding Figure 4 and EV3, regarding the differences in tubulin staining. Please provide new stainings, if required.

- **New staining has now been included for the relevant studies (Figure EV5).**

- Finally, please comment on the differences between cold-soluble and -insoluble tubulin.

- **We believe this has been clarified by graphing the data in a different way (Fig. 4B), and by changing the wording in the figure legend.**

Browsing through the manuscript myself, I noticed a few editorial things that we need before we can proceed with the acceptance of your study.

- You have submitted your manuscript as Scientific Report. This article type can only contain up to 5 figures. Since you currently have 6 figures, I suggest to change it to a full Article. In this case, the Results and Discussion section have to be separated.

- **We have moved some panels to Expanded View so that figures 5 and 6 could be combined.**

- Statistics: I noticed that in some cased the number of individual experiments was 2 (n=2, e.g., Fig 3, 4, 5, and 6). Please note that in these cases the application of statistical tests is not appropriate due to the small sample size. Please review all figure panels and apply statistical test only to data that is obtained from at least 3 three independent experiments.

- **This has been corrected throughout.**

- The Author Checklist will be published alongside your manuscript. Therefore, please update the information in the section F- Data Accessibility.

- **This has been added to the checklist, and changed in the materials and methods section.**

- Please provide a running title of max. 40 characters incl. spaces on the first page of the manuscript.

- **This has been added.**

- Please provide up to five keywords.

- **These have been provided.**

- Please update the reference list to match the EMBO reports style. The abbreviation 'et al' should be used if there are more than ten authors and the first ten author names should be listed. You can download the respective EndNot file from our Guide to Authors

- **This has been corrected.**

EMBO PRESS

YOU MUST COMPLETE ALL CELLS WITH A PINK BACKGROUND ➔

PLEASE NOTE THAT THIS CHECKLIST WILL BE PUBLISHED ALONGSIDE YOUR PAPER

Corresponding Author Name: Stephen D Robinson
Journal Submitted to: EMBO Reports
Manuscript Number: 2017-44578V2

Reporting Checklist For Life Sciences Articles (Rev. June 2017)

This checklist is used to ensure good reporting standards and to improve the reproducibility of published results. These guidelines are consistent with the Principles and Guidelines for Reporting Preclinical Research issued by the NIH in 2014. Please follow the journal's authorship guidelines in preparing your manuscript.

A- Figures
1. Data
The data shown in figures should satisfy the following conditions:

- ➔ the data were obtained and processed according to the field's best practice and are presented to reflect the results of the experiments in an accurate and unbiased manner.
- ➔ figure panels include only data points, measurements or observations that can be compared to each other in a scientifically meaningful way.
- ➔ graphs include clearly labeled error bars for independent experiments and sample sizes. Unless justified, error bars should not be shown for technical replicates.
- ➔ if n< 5, the individual data points from each experiment should be plotted and any statistical test employed should be justified
- ➔ Source Data should be included to report the data underlying graphs. Please follow the guidelines set out in the author ship guidelines on Data Presentation.

2. Captions

Each figure caption should contain the following information, for each panel where they are relevant:

- ➔ a specification of the experimental system investigated (eg cell line, species name).
- ➔ the assay(s) and method(s) used to carry out the reported observations and measurements
- ➔ an explicit mention of the biological and chemical entity(ies) that are being measured.
- ➔ an explicit mention of the biological and chemical entity(ies) that are altered/varied/perturbed in a controlled manner.

- ➔ the exact sample size (n) for each experimental group/condition, given as a number, not a range;
- ➔ a description of the sample collection allowing the reader to understand whether the samples represent technical or biological replicates (including how many animals, litters, cultures, etc.).
- ➔ a statement of how many times the experiment shown was independently replicated in the laboratory.
- ➔ definitions of statistical methods and measures:
  - common tests, such as t-test (please specify whether paired vs. unpaired), simple χ2 tests, Wilcoxon and Mann-Whitney tests, can be unambiguously identified by name only, but more complex techniques should be described in the methods section;
  - are tests one-sided or two-sided?
  - are there adjustments for multiple comparisons?
  - exact statistical test results, e.g., P values = x but not P values < x;
  - definition of 'center values' as median or average;
  - definition of error bars as s.d. or s.e.m.

Any descriptions too long for the figure legend should be included in the methods section and/or with the source data.

In the pink boxes below, please ensure that the answers to the following questions are reported in the manuscript itself. Every question should be answered. If the question is not relevant to your research, please write NA (non applicable). We encourage you to include a specific subsection in the methods section for statistics, reagents, animal models and human subjects.

USEFUL LINKS FOR COMPLETING THIS FORM

http://www.antibodypedia.com
http://1degreebio.org
http://www.equator-network.org/reporting-guidelines/improving-bioscience-research-repo
http://grants.nih.gov/grants/olaw/olaw.htm
http://www.mrc.ac.uk/Ourresearch/Ethicsresearchguidance/Useofanimals/index.htm
http://ClinicalTrials.gov
http://www.consort-statement.org
http://www.consort-statement.org/checklists/view/32-consort/66-title
http://www.equator-network.org/reporting-guidelines/reporting-recommendations-for-tum
http://datadryad.org
http://figshare.com
http://www.ncbi.nlm.nih.gov/gap
http://www.ebi.ac.uk/ega
http://biomodels.net/
http://biomodels.net/miriam/
http://jjj.biochem.sun.ac.za
http://oba.od.nih.gov/biosecurity/biosecurity_documents.html
http://www.selectagents.gov/

B- Statistics and general methods

Please fill out these boxes ➔ (Do not worry if you cannot see all your text once you press return)

1.a. How was the sample size chosen to ensure adequate power to detect a pre-specified effect size?	Sample size for mass spectrometry experiments were chosen based on best practise in the field and recommendations of the mass spectrometry service.
1.b. For animal studies, include a statement about sample size estimate even if no statistical methods were used.	Necessary sample size was estimated using previous data obtained using the same tumour model in the same colony of mice. Experience of this model in Steri et al (2013) and Ellison et al (2015) allowed us to minimise the number of mice used in line with 3R's principles.
2. Describe inclusion/exclusion criteria if samples or animals were excluded from the analysis. Were the criteria pre-established?	Adhesome samples were excluded based on silver staining band patterns of a fraction of the sample post precipitation but pre mass spectrometry analysis. These criteria were identified during optimisation of the technique for endothelial cells. For tumour experiments, animals were excluded if the tumour was not subcutaneously placed (e.g. injection into peritoneum or skeletal muscle).
3. Were any steps taken to minimize the effects of subjective bias when allocating animals/samples to treatment (e.g. randomization procedure)? If yes, please describe.	Mouse colonies were managed so litters were born that were all Itgb3 floxed with half cre positive and half cre negative allowing the use of littermate controls. Tumours were implanted into all mice and genotype was confirmed after tumour harvest/measurements.
For animal studies, include a statement about randomization even if no randomization was used.	See above
4.a. Were any steps taken to minimize the effects of subjective bias during group allocation or/and when assessing results (e.g. blinding of the investigator)? If yes please describe.	Yes, see above; vessel counts were performed blind of genotype.
4.b. For animal studies, include a statement about blinding even if no blinding was done	See above
5. For every figure, are statistical tests justified as appropriate?	Yes - significant analysis of microarrays (SAM) was chosen as best practise in the mass spectrometry field in dealing with the multiple comparison problem common in "omics" studies. Due to this recommendation, SAM analysis was available natively in Perseus (a mass spectrometry statistics package) which was used for this study. Unpaired, two-tailed Student's t-tests were performed for all other analyses.
Do the data meet the assumptions of the tests (e.g., normal distribution)? Describe any methods used to assess it.	Whilst non-normal distributions can be accounted for in Perseus by using alternative T test methods during the SAM analysis, it was possible check for the distribution of protein abdundances and apply the appropriate test
Is there an estimate of variation within each group of data?	Where n number is sufficiently large, data normality is tested via D'Agostino-Pearson normality test (omnibus K2).

Is the variance similar between the groups that are being statistically compared?	Yes.

C- Reagents

6. To show that antibodies were profiled for use in the system under study (assay and species), provide a citation, catalog number and/or clone number, supplementary information or reference to an antibody validation profile. e.g., Antibodypedia (see link list at top right), 1DegreeBio (see link list at top right).	anti-alpha-tubulin (Abcam 52866), anti-paxillin (Abcam 32084), anti-talin (Sigma T3287), anti-neuropilin-1 (R&D systems SF566), anti-CD31 (R&D systems AF3628).
7. Identify the source of cell lines and report if they were recently authenticated (e.g., by STR profiling) and tested for mycoplasma contamination.	Endothelial "cell lines" were produced in-house. Not tested within the last three months for mycoplasma contamination, but historically negative. CMT19T lung carcinoma cells originally from Cancer Research UK cell repository. Not recently STR profiled. Lab practice is to freeze down large banks of cells that are mycoplasma tested. A fresh vial is thawed for each in vivo experiment. When frozen stock runs low, it is replenished with a mycoplasma tested batch.

* for all hyperlinks, please see the table at the top right of the document

D- Animal Models

8. Report species, strain, gender, age of animals and genetic modification status where applicable. Please detail housing and husbandry conditions and the source of animals.	Mice on a mixed C57BL/6:129Sc background; littermate controls used for all studies. Mice were 8-12 weeks old at time of CMT19T cell implantation. Mice were floxed for beta3-integrin and either Cre-neg or Cre-pos for Tie1.Cre. Mice are housed in individually vented cages, no more than 5 per cage. Mice were bred in-house.
9. For experiments involving live vertebrates, include a statement of compliance with ethical regulations and identify the committee(s) approving the experiments.	We confirm compliance with ethical regulations governing the use of these animals, as stipulated by our local Animal Welfare Committee, and our Home Office Project Licence.
10. We recommend consulting the ARRIVE guidelines (see link list at top right) (PLoS Biol. 8(6), e1000412, 2010) to ensure that other relevant aspects of animal studies are adequately reported. See author guidelines, under 'Reporting Guidelines'. See also: NIH (see link list at top right) and MRC (see link list at top right) recommendations. Please confirm compliance.	We confirm compliance

E- Human Subjects

11. Identify the committee(s) approving the study protocol.	N/A
12. Include a statement confirming that informed consent was obtained from all subjects and that the experiments conformed to the principles set out in the WMA Declaration of Helsinki and the Department of Health and Human Services Belmont Report.	N/A
13. For publication of patient photos, include a statement confirming that consent to publish was obtained.	N/A
14. Report any restrictions on the availability (and/or on the use) of human data or samples.	N/A
15. Report the clinical trial registration number (at ClinicalTrials.gov or equivalent), where applicable.	N/A
16. For phase II and III randomized controlled trials, please refer to the CONSORT flow diagram (see link list at top right) and submit the CONSORT checklist (see link list at top right) with your submission. See author guidelines, under 'Reporting Guidelines'. Please confirm you have submitted this list.	N/A
17. For tumor marker prognostic studies, we recommend that you follow the REMARK reporting guidelines (see link list at top right). See author guidelines, under 'Reporting Guidelines'. Please confirm you have followed these guidelines.	N/A

F- Data Accessibility

18: Provide a "Data Availability" section at the end of the Materials & Methods, listing the accession codes for data generated in this study and deposited in a public database (e.g. RNA-Seq data: Gene Expression Omnibus GSE39462, Proteomics data: PRIDE PXD000208 etc.) Please refer to our author guidelines for 'Data Deposition'.<br><br>Data deposition in a public repository is mandatory for:<br>a. Protein, DNA and RNA sequences<br>b. Macromolecular structures<br>c. Crystallographic data for small molecules<br>d. Functional genomics data<br>e. Proteomics and molecular interactions	The mass spectrometry proteomics data have been deposited to the ProteomeXchange Consortium via the PRIDE partner repository with the dataset identifier PXD008591
19. Deposition is strongly recommended for any datasets that are central and integral to the study; please consider the journal's data policy. If no structured public repository exists for a given data type, we encourage the provision of datasets in the manuscript as a Supplementary Document (see author guidelines under 'Expanded View' or in unstructured repositories such as Dryad (see link list at top right) or Figshare (see link list at top right).	
20. Access to human clinical and genomic datasets should be provided with as few restrictions as possible while respecting ethical obligations to the patients and relevant medical and legal issues. If practically possible and compatible with the individual consent agreement used in the study, such data should be deposited in one of the major public access-controlled repositories such as dbGAP (see link list at top right) or EGA (see link list at top right).	
21. Computational models that are central and integral to a study should be shared without restrictions and provided in a machine-readable form. The relevant accession numbers or links should be provided. When possible, standardized format (SBML, CellML) should be used instead of scripts (e.g. MATLAB). Authors are strongly encouraged to follow the MIRIAM guidelines (see link list at top right) and deposit their model in a public database such as Biomodels (see link list at top right) or JWS Online (see link list at top right). If computer source code is provided with the paper, it should be deposited in a public repository or included in supplementary information.	

G- Dual use research of concern

22. Could your study fall under dual use research restrictions? Please check biosecurity documents (see link list at top right) and list of select agents and toxins (APHIS/CDC) (see link list at top right). According to our biosecurity guidelines, provide a statement only if it could.	No
